# Supplementary material for: Two-photon activated precision molecular photosensitizer targeting mitochondria
Source: Commun Chem. 2021 Oct 7;4:142. doi: 10.1038/s42004-021-00581-4 (PMC9814857; doi:10.1038/s42004-021-00581-4)
Supplement: Supplementary file 1 — Description of Additional Supplementary Files [file 42004_2021_581_MOESM1_ESM.pdf]

Description of Additional Supplementary Files

File Name: Supplementary Movie 1

Description:

Mitochondria damage inflicted by a quinolizinium cation (Q2) under multiphoton irradiation at 800 nm.

No damage is observed when irradiation is done in the absence of the photosensitizer quinolizinium (not shown).

File Name: Supplementary Movie 2

Description:

Mitochondria damage inflicted by a quinolizinium cation (Q2) under multiphoton irradiation at 800 nm. No damage is observed when irradiation is done in the absence of the photosensitizer quinolizinium (not shown).
