# Supplementary material for: Two-photon activated precision molecular photosensitizer targeting mitochondria
Source: Commun Chem. 2021 Oct 7;4:142. doi: 10.1038/s42004-021-00581-4 (PMC9814857; doi:10.1038/s42004-021-00581-4)
Supplement: Supplementary file 2 — Supplementary Information [file 42004_2021_581_MOESM2_ESM.pdf]

Supplementary information

Two-photon activated precision molecular photosensitizer targeting mitochondria

Inês F. A. Mariz,[1] Sandra N. Pinto,[2,3] Ana M. Santiago,[1] José M. G. Martinho,[1] Javier Recio,[4] Juan J. Vaquero,[4] Ana M. Cuadro,*[4] and Ermelinda Maçôas,*[1]

1 Dr. Inês F. A. Mariz, Dr. Ana M. Santiago, Prof. José M. G. Martinho and Dr. Ermelinda Maçôas
Centro de Química Estrutural and Institute of Molecular Sciences (IMS), Instituto Superior Técnico, Universidade de Lisboa, 1049-001 Lisboa, Portugal.
E-mail: ermelinda.macoas@tecnico.ulisboa.pt

2 Dr. Sandra N. Pinto, Institute of Bioengineering and Bioscience (IBB), and Institute for Health and Bioeconomy (i4HB), Instituto Superior Técnico, Universidade de Lisboa, 1049-001 Lisboa, Portugal and

3 Dr. Sandra N. Pinto, Associate Laboratory - Institute for Health and Bioeconomy (i4HB), Instituto Superior Técnico, Universidade de Lisboa, Av. Rovisco Pais, 1049-001 Lisboa, Portugal.

4 Dr. Javier Recio, Prof. Juan J. Vaquero and Prof. Ana M. Cuadro Departamento de Química Orgánica y Química Inorgánica, Universidad de Alcalá, (IRYCIS); 28871-Alcalá de Henares, Madrid, Spain. Email: ana.cuadro@uah.es

Contents

1. SUPPLEMENTARY METHODS 2

1.1 Materials and Reagents 2

1.2 Synthesis of quinolizinium 3

1.2.1. Synthesis dipolar D-π-A+ compounds **Q2** and **Q3**. *Knoevenagel reaction.* 3

1.3.2 Synthesis of quadrupolar D-π-A+-π-D compounds **Qe2** and **Qe3**. 3

1.3 Synthesis of benzimidazolium derivatives 5

1.3.1 Synthesis of starting materials: 5

1.3.2 Synthesis of dipolar D-π-A+ benzimidazoliums (**B2** and **B4**). 6

1.3.3. Synthesis of quadrupolar D-π-A+-A+-π-D bisbenzimidazoliums (**Be2** and **Be4**). 6

1.4 ^{1}H and ^{13}C NMR for all new compounds reported 8

2. OPTICAL PROPERTIES 16

3. CELL CULTURE AND STAINING 22

4. PHOTOIRRADIATION EFFECT 25

5. SINGLET OXYGEN QUANTUM YIELD 30

6. SUPPLEMENTARY REFERENCES 30

1. Supplementary Methods

1.1 Materials and Reagents

Infrared spectra were recorded on KBr pellets. Reactions were monitored by thin-layer chromatography (TLC) using TLC silica gel-coated aluminium plates 60F-254 (Merck). Column chromatography was performed using Merck silica gel 60, 0.040–0.063 mm (230–400 mesh). NMR spectroscopy was performed on Varian UNITY-Plus 300 MHz ^{1}H (and 75 MHz ^{13}C) or VNMRS-500 MHz ^{1}H (and 125 MHz ^{13}C) spectrometers equipped with a cryoprobe. Chemical shifts were reported as δ values (ppm) and coupling constants (J) in Hz. The mass spectra (MS) as ESI^{+} and HRMS masses were recorded on a Thermo Scientific TSQ Quantum LC/MS equipped with an ESI ionization source and a TOF detector. All starting materials, namely $Pd(OAc)_2$, 4-methoxy- and 4-*N*,*N*-dimethylamino-benzaldehyde, and the corresponding *o*-phenylenediamine and 1,1'-bifenyl-3,3',4,4'-tetraamine were purchased from Aldrich and were used without further purification. DMF, and Et3N were distilled over activated molecular sieves. The 2-methylquinolizinium and 2,8-dimethylquinolizinium salts were obtained by adapting described methods and changing the counterion (see ref. 1). The 1,2,3-trimethylbenzimidazolium iodide **B'** was prepared by alkylation of methyl benzimidazole with methyl iodide in the presence of sodium hydride. Analogous procedure was applied to obtain the salt **Be'**. Preparation of (E)-2-(arylvinyl)quinolizinium hexafluorophosphates by Heck reaction and Knoevenagel reaction followed procedures described in refs. 2, 3, and 4. 4-[(1E)-2-(4-methoxy/dimethylamino)phenyl)ethenyl] benzaldehyde was prepared following the literature procedure (see refs. 5 and 6). In the following a more detailed description of synthetic procedure, as well as the NMR spectra of the synthetized compounds, are given.

1.2 Synthesis of quinolizinium

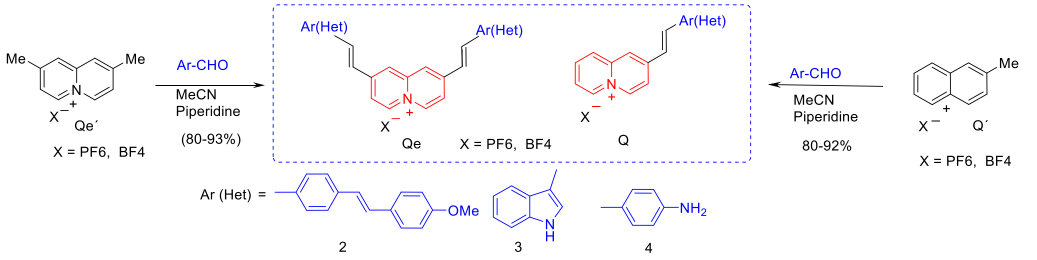

Scheme S1. Procedure to obtain quinolizinium-based dipolar and quadrupolar molecules

1.2.1. Synthesis dipolar D-π-A$^+$ compounds **Q2** and **Q3**. *Knoevenagel reaction.*

Piperidine (41 µL, 0.41 mmol, 1.2 equiv.) was added to a refluxing acetonitrile solution of 2-methylquinolizinium hexafluorophosphate (100 mg, 0.34 mmol) and aryl/heteroaryl aldehyde (0.41 mmol, 1.2 equiv.). The mixture was refluxed for 2 hours. After cooling to room temperature, diethyl ether (30 mL) was added. The resulting precipitate was filtered, washed with ether, and dried in vacuo to give (E)-2-(arylvinyl)quinolizinium Hexafluorophosphates. Analytically pure sample was obtained by recrystallization from acetonitrile-ethanol.

2-[(*E*)-2-(4-((*E*)-2-(4-metoxifenil)vinil)fenil)vinil]quinolizinium hexafluorophosphate (Q2). From 2-methylquinolizinium hexafluorophosphate (0.100 g, 0.346 mmol) and 4-[(1E)-2-(4-methoxyphenyl) ethenyl]-benzaldehyde (0.124 g, 0.52 mmol, 1,2 equiv.) in 3 mL of dry acetonitrile, following the general procedure, 133 mg (75%) of **Q2** were obtained as a dark red solid. IR (KBr), $\tilde{\upsilon}_{max}$ (cm^{-1}): 3118, 2998, 1646, 1662, 1592, 1515, 1456, 1407, 1306, 1251, 1176, 1152, 968, 840, 557.^{1}H-RMN (500 MHz, DMSO-*d$_6$*) δ(ppm): 9.26 (d, *J* = 7.2 Hz, 1H), 9.19 (d, *J* = 6.8 Hz, 1H), 8.52 (d, *J* = 2.0 Hz, 1H), 8.45 – 8.37 (m, 2H), 8.29 (d, *J* = 1.3 Hz, 1H), 7.97 (d, *J* = 1.4 Hz, 1H), 7.93 (d, *J* = 16.3 Hz, 1H), 7.76 (d, *J* = 8.1 Hz, 2H), 7.69 (d, *J* = 8.1 Hz, 2H), 7.62 – 7.53 (m, 3H), 7.33 (d, *J* = 16.4 Hz, 1H), 7.16 (d, *J* = 16.4 Hz, 1H), 6.97 (d, *J* = 1.9 Hz, 2H), 3.79 (s, 3H). ^{13}C NMR (75 MHz, DMSO-*d$_6$*) δ(ppm): 159.1, 144.9, 142.6, 139.0, 138.0, 136.6, 136.4, 136.3, 134.0, 129.4, 129.3, 128.1, 127.9, 126.7, 126.6, 125.2, 123.1, 122.6, 120.1, 114.1, 55.2. HRMS, (ESI-TOF) m/z Calcd. for $C_{26}H_{22}NO$ [M]$^+$: 364.1696, Found: 364.1696.

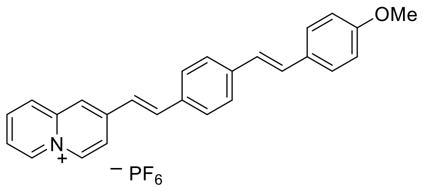

2-[(*E*)-2-(1H-indol-3-il)vinil]quinolizinium hexafluorophosphate (Q3). From 2-methylquinolizinium hexafluorophosphate (0.100 g, 0.346 mmol) and 1H-indole-3-carboxaldehyde (75 mg, 0.52 mmol), in 3 mL of dry acetonitrile, following the general procedure, 126 mg (86%) of **Q3** were obtained as an orange solid. IR (KBr), $\tilde{\upsilon}_{max}$ (cm^{-1}): 3421, 1624, 1526, 1456, 1409, 1253, 1153, 1104, 955, 903, 840, 745, 558. ^{1}H NMR (300 MHz, DMSO-*d$_6$*) δ(ppm): 11.83 (s, 1H), 9.12 (d, *J* = 7.2 Hz, 1H), 9.05 (d, *J* = 6.8 Hz, 1H), 8.42 (s, 1H), 8.35 (d, *J* = 7.2 Hz, 1H), 8.26 (d, *J* = 8.6 Hz, 1H), 8.22 – 8.08 (m, 3H), 7.93 (s, 1H), 7.81 (t, *J* = 6.9 Hz, 1H), 7.50 (d, *J* = 8.8 Hz, 1H), 7.34 (d, *J* = 16.2 Hz, 1H), 7.28 – 7.15 (m, 2H). 13C NMR (75 MHz, DMSO-*d$_6$*) δ(ppm): 147.0, 142.6, 137.3, 136.0, 135.9, 135.9, 133.7, 130.8, 126.2, 124.8, 122.6, 121.3, 120.8, 120.1, 120.0, 119.6, 117.5, 113.4, 112.4. HRMS, (ESI-TOF) m/z Calcd. for $C_{19}H_{15}N_2$ [M]$^+$: 271.1230, Found: 271.1204.

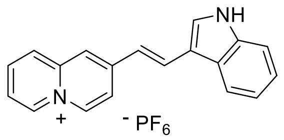

1.3.2 Synthesis of quadrupolar D-π-A$^+$-π-D compounds **Qe2** and **Qe3**.

Synthesis of (*E*,*E*)-2,8-Bis(aryl/heteroarylvinyl)quinolizinium salts by *Knoevenagel reaction.* *Piperidine (2 equiv.) was added to a refluxing acetonitrile solution of 2,8-dimethylquinolizinium hexafluorophosphate (1 equiv.) and arylaldehyde (2.5 equiv.). The mixture was refluxed for 6 hours. After completion of the reaction, the mixture was allowed to cool down to room temperature, then diethyl ether was added. The precipitate was collected by filtration and washed with diethyl ether. The solid residue was dried under vacuum to give (E,E)-2,8-bis(aryl/heteroarylvinyl)quinolizinium hexafluorophosphates. Analytically pure sample was obtained by recrystallization from acetonitrile-ethanol.*

2,8-bis[(1*E*)-2-[4-[(1*E*)-2-(4-methoxyphenyl)ethenyl]phenyl]ethenyl]quinolizinium hexafluorophosphate ($Qe2.PF_6^-$). From 2,8-dimethylquinolizinium hexafluorophosphate (100 mg, 0.33 mmol) and 4-[(1E)-2-(4-methoxyphenyl) etenyl]-benzaldehyde (196 mg, 0,825 mmol, 2.5 equiv.), following the general procedure, 245 mg (98%) of **$Qe2.PF_6^-$** were obtained as an orange solid. IR (KBr), $\nu_{máx}$ (cm^{-1}) 1644, 1618, 1590, 1514, 1451, 1173, 842.4. ^1H NMR (500 MHz, DMSO-d_6) δ(ppm): 9.10 (d, *J* = 7.4 Hz, 2H), 8.34 (s, 2H), 8.27 (d, *J* = 7.3 Hz, 2H), 7.89 (d, *J* = 16.3 Hz, 2H), 7.74 (s, 4H), 7.69 (d, *J* = 8.1 Hz, 4H), 7.61 – 7.53 (m, 6H), 7.33 (d, *J* = 16.4 Hz, 2H), 7.16 (d, *J* = 16.4 Hz, 2H), 6.97 (d, *J* = 8.8 Hz, 4H), 3.79 (s, 6H). ^{13}C NMR (75 MHz, DMSO-d_6) δ(ppm): 159.3, 145.0, 143.0, 139.2, 137.9, 136.2, 134.3, 129.5, 128.3, 128.1, 126.8, 125.4, 123.4, 122.9, 118.9, 114.3, 55.2. HRMS, (ESI-TOF) m/z Calcd. for $C_{43}H_{36}NO_2$ $[M]^+$: 598.2741, Found: 598.2734. MS (ESI^-) *m/z* (relative intensity, %): 145 $[M+1]^+$ (100).

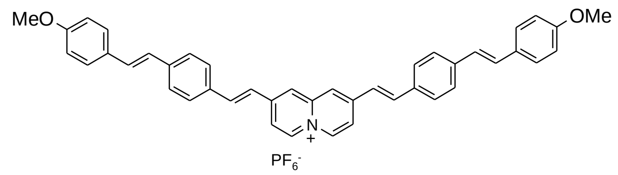

2,8-bis[(1E)-2-[4-[(1E)-2-(4-methoxyphenyl)ethenyl] phenyl]ethenyl] quinolizinium tetrafluoroborate ($Qe2.BF_4^-$). From 2, 8-dimethylquinolizinium tetrafluoroborate (44 mg, 0.18 mmol) and 4-[(1E)-2-(4-methoxyphenyl) etenyl]-benzaldehyde (107 mg, 0.45 mmol, 2.5 equiv.), following the general procedure, 40 mg (32%) of **$Qe2.BF_4^-$** were obtained as a red solid. IR (KBr) ν_{max} (cm^{-1}) 3432, 2924, 2853, 2360, 1733, 1644, 1619, 1590, 1515, 1451, 1425, 1304, 1252, 1173, 1063, 1032, 962, 831, 605, 539. ^1H-RMN (500 MHz, DMSO-d_6) δ (ppm): 9.05 (d, *J*= 7.2Hz, 2H), 8.27 (s, 2H), 8.22 (d, *J*= 7.2Hz, 2H), 7.82 (d, *J*= 16.1Hz, 2H), 7.70 (d, *J*= 8.4Hz, 4H), 7.64 (d, J= 8.4Hz, 4H), 7.54 (d, J= 8.8Hz, 4H), 7.50 (d, J= 16.1 Hz, 2H), 7.29 (d, J= 16.3 Hz, 2H), 7.11 (d, *J*= 16.3 Hz, 2H), 6.93 (d, *J*= 8.8 Hz, 4H). ^{13}C-RMN (75 MHz, DMSO-d_6) δ (ppm): 159.7, 145.4, 143.4, 139.6, 138.3, 136.6, 134.7, 129.9, 128.7, 128.5, 127.2, 125.8, 123.9, 123.3, 119.4, 115.1, 114.7, 55.6. MS (ESI^+) *m/z* (relative intensity, %): 624 (M^+, 100), 625 (M+1, 50). MS (ESI^-) *m/z* (relative intensity, %): 87 $[M+1]^+$ (100).

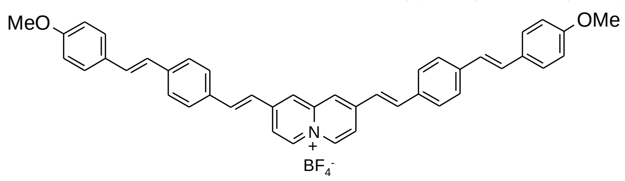

2,8-bis[(1*E*)-2-(1*H*-indole-3-yl)ethenyl]quinolizinium hexafluorophosphate ($Qe3.PF_6^-$)[7] From 2,8-dimethyl quinolizinium hexafluorophosphate (80.0 mg, 0.264 mmol) and 1*H*-indole-3-carboxaldehyde (96.0 mg, 0.660 mmol, 2.5 equiv.), following the general procedure, 113 mg (77%) of **$Qe3.PF_6^-$** were obtained as a dark red solid. IR (KBr) ν_{max} (cm^{-1}) 3400, 3118, 1647, 1607, 1523, 1451, 1403, 1310, 1248, 1165, 1133, 1108, 957, 838, 748, 651, 610, 558. ^1H NMR (500 MHz, DMSO-d_6) δ(ppm): 11.83 (s, 2H), 8.93 (d, *J* = 7.2 Hz, 2H), 8.22 – 8.12 (m, 6H), 8.09 (d, *J* = 16.1 Hz, 2H), 7.94 (s, 2H), 7.51 (d, *J* = 7.6, 1.6 Hz, 2H), 7.36 (d, *J* = 16.2 Hz, 2H), 7.29 – 7.21 (m, 4H). ^{13}C NMR (75 MHz, DMSO) δ(ppm): 146.2, 143.0, 137.2, 135.3, 132.8, 130.5, 124.8, 122.5, 120.6, 120.1, 119.8, 117.86, 117.3, 113.4, 112.3. HRMS, (ESI-TOF) m/z Calcd. for $C_{29}H_{22}N_3$ $[M]^+$: 412.1808, Found: 412.1790. MS (ESI^+) *m/z* (relative intensity, %): 412 $[M]^+$ (100), 413 $[M+1]^+$ (30). MS (ESI^-) *m/z* (relative intensity, %): 145 $[M+1]^+$ (100).

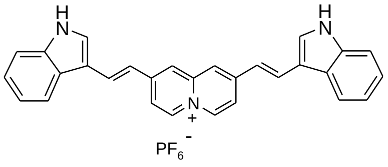

2,8-bis[(1*E*)-2-(1*H*-indole-3-yl)ethenyl]quinolizinium tetrafluoroborate ($Qe3.BF_4^-$). From 2,8-dimethyl quinolizinium tetrafluoroborate (66 mg, 0.27 mmol) and 1H-indole-3-carboxaldehyde (0.100 g, 0.69 mmol, 2.5 equiv.), in acetonitrile (5mL) following the general procedure, 124 mg (92%) of **$Qe3.BF_4^-$** were obtained as a red solid. IR (KBr), ῡmax (cm^{-1}): 3398, 1608, 1504, 1179, 1044, 882, 575. ^1H NMR (300 MHz, DMSO-d_6) δ(ppm): 11.94 (s, 2H), 8.93 (d, 2H, J= 6.9), 8.17 – 8.15 (m, 6H), 8.09 (d, 2H, J= 16.4), 7.93 (s, 2H), 7.49 (d, 2H, J= 6,9), 7.35 (d, 2H, J= 16.4), 7.27 – 7.20(m, 4H). ^{13}C NMR (75 MHz, DMSO) δ(ppm): 146.5, 140.1, 137.5, 135.6, 133.1, 130.8, 125.0, 122.8, 120.9, 120.3, 120.1, 118.1, 117.6, 113.6, 112.6. HRMS, (ESI-TOF) m/z Calcd. for $C_{29}H_{22}N_3$ [M+]: 412.1808. Found:[M]+ 412.1798. MS (ESI^-) *m/z* (relative intensity, %): 87 $[M+1]^+$ (15), 91 (68).

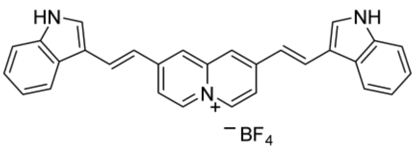

1.3 Synthesis of benzimidazolium derivatives

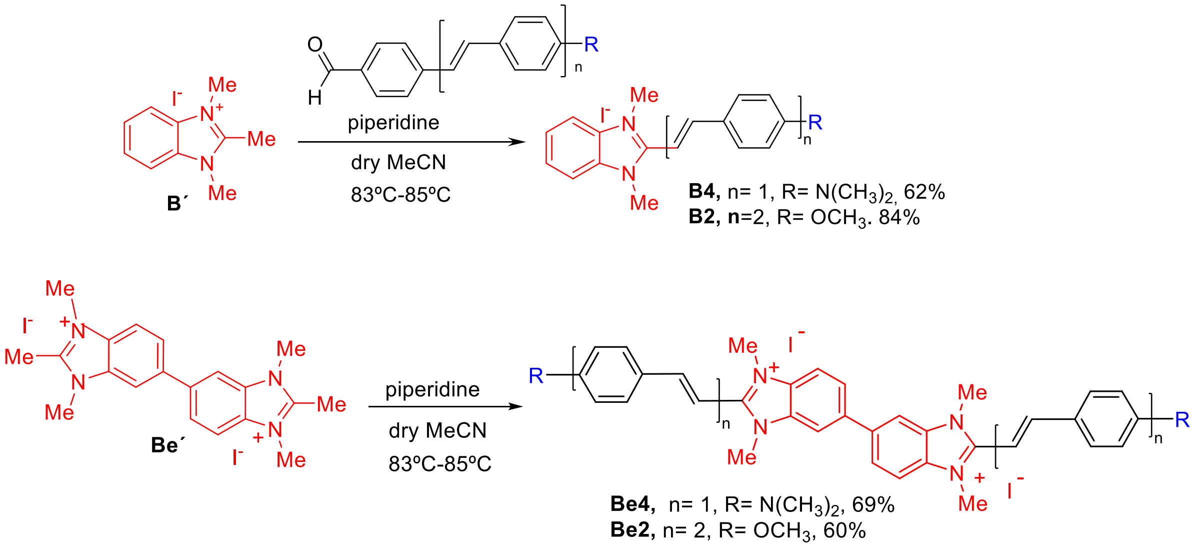

Scheme S2. a) Synthesis of benzimidazolium derivatives: dipolar **B2** and **B4** with D-π-A^+ structure, and b) Quadrupolar **Be2** and **Be4** with D-π-A^+-A^+-π-D structure.

1.3.1 Synthesis of starting materials:

General procedure of alkylation reaction[8]

NaH (1.5 equiv. or 3 equiv.) was added to a solution of the corresponding benzimidazole or bisbenzimidazole derivative (1 equiv.) in a sealed tube in DMF (10 mL), and MeI (24 equiv). The reaction mixture was stirred at 110 ° C for 20 h, and then cooled to room temperature. The solid formed was isolated by vacuum filtration and washed with ethyl acetate to obtain the final product.

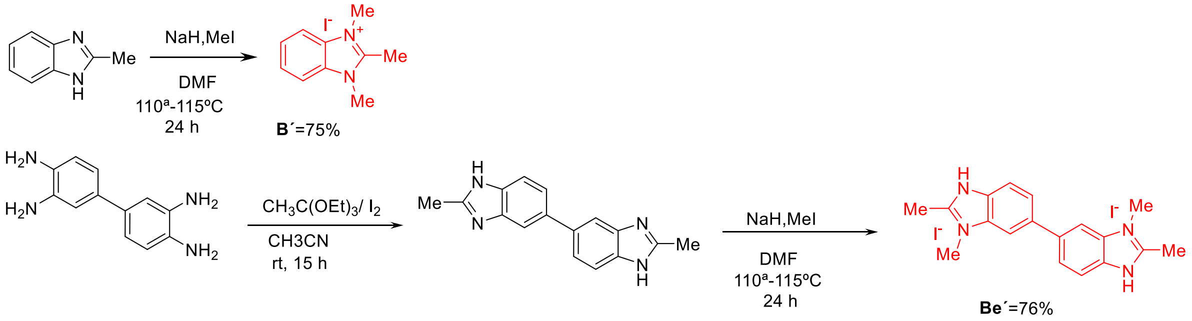

Scheme S3. Synthesis of precursors **B’** and **Be’**.

General procedure of Wittig reaction[5]

To a solution of triphenylphosphonium bromide (1.1 equiv.) and sodium tert-butoxide (1.2 equiv.) in dry THF (10 mL) at 0°C under Argon atmosphere, a solution of the corresponding aldehyde derivative (1 equiv.) in dry THF (10 mL) was added via a cannula. The reaction mixture was stirred for 48 h at room temperature, the mixture, was extracted with ethyl acetate and washed with brine. The organic layers were dried over anhydrous Mg_2SO_4, filtered, and concentrated under reduced pressure. The resulting oil was purified by flash silica chromatography using hexane/AcOEt (9: 1) as the solvent.

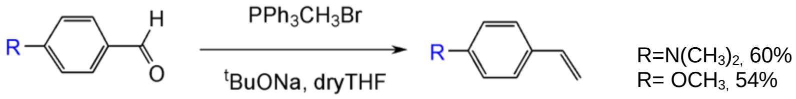

Scheme S4. Synthesis of precursors methoxy/dimethylamino-benzaldehydes via Witting reactions.

General procedure Heck reaction[6]

A solution of the corresponding vinyl derivative in dry DMF (10 mL) was added to a solution of 4-bromobenzaldehyde (1 equiv.), potassium carbonate (2 equiv.), tetrabutylammonium bromide (1 equiv.) and Pd $(OAc)_2$ (1%)) under an argon atmosphere in dry DMF (10 mL). The reaction mixture was stirred for 15 h at 110 °C. The mixture was extracted with CH_2Cl_2 and washed with brine. The organic layers were dried over anhydrous Mg_2SO_4, filtered, and concentrated under reduced pressure. The resulting solid was purified by flash silica chromatography using hexane/AcOEt (8:2) as the solvent.

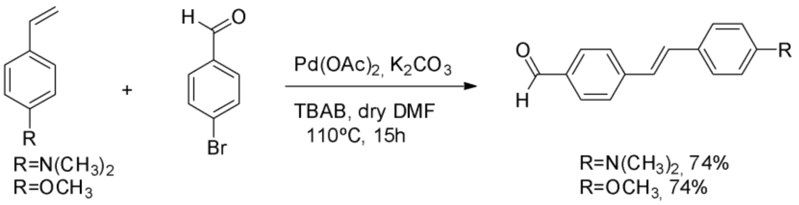

Scheme S5. Synthesis of precursors 4-[(1E)-2-(4-methoxy-phenyl)ethenyl]-benzaldehyde via Heck reactions.

1.3.2 Synthesis of dipolar D-π-A+ benzimidazoliums (B2 and B4).

Piperidine (1 equiv.) was added to a refluxing solution of 1,2,3-trimethylbenzimidazolium iodide (1 equiv.) and the corresponding aldehyde (1.2 equiv.) in dry MeCN and the reaction mixture was heated at 80-85 °C for 3-15 h. After completion of the reaction, the mixture was allowed to cool down at room temperature. The solid was filtered off, washed with ether, and dried in vacuo to give the final product.

2-[(*E*)-2-(4-((*E*)-2-(4-(metoxifenil)etenil)fenil)etenil]-1,3-dimetilbenzimidazolium iodide (B2). From

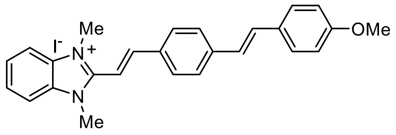

1,2,3-Trimethyl-1H-benzimidazol-3-ium iodide (108 mg, 0.37 mmol) 6, 4-[(1E)-2-(4-methoxyphenyl) etenyl]-benzaldehyde (100 mg, 0.45 mmol, 2.5 equiv.), and piperidine (37 µL, 0.37 mmol), in acetonitrile (5mL), following the general procedure, 160 mg (84%) of **B2** were obtained as a light yellow solid. IR, (KBr), $\tilde{\upsilon}_{máx}$ (cm^{-1}) 2957.0, 2831.5, 1628.5, 1606.9, 1590.3, 1509.2, 1457.5, 1173.0, 834.8. ^{1}H NMR (500 MHz, DMSO-d_6) δ (ppm): 8.09 – 8.02 (m, 2H), 7.94 (d, J = 8.0 Hz, 2H), 7.82 (d, J = 16.7 Hz, 1H), 7.74 (d, J = 8.0 Hz, 2H), 7.72 – 7.67 (m, 2H), 7.60 (d, J = 8.3 Hz, 2H), 7.56 (d, J = 16.7 Hz, 1H), 7.40 (d, J = 16.4 Hz, 1H), 7.19 (d, J = 16.4 Hz, 1H), 6.98 (d, J = 8.3 Hz, 2H), 4.16 (s, 6H), 3.79 (s, 3H). ^{13}C NMR (75 MHz, DMSO) δ (ppm): 159.1, 148.1, 145.8, 140.2, 132.86, 131.8, 130.0, 129.20, 128.9, 18.0, 126.5, 126.2, 125.1, 114.1, 112.8, 106.9, 55.2, 32.9. HRMS, (ESI-TOF) m/z Calcd. for $C_{26}H_{25}N_2O$ $[M]^+$: 381.1961, Found: 381.1967.

2-{2-[4-(dimethylamino)phenyl]vinyl}-1,3-dimethyl-1H-3,1-benzimidazolium iodide (B4).[9] From 1,2,3-

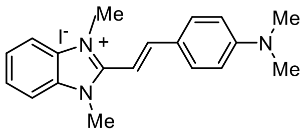

Trimethyl-1H-benzimidazol-3-ium iodide (0.58 g, 2 mmol) *p*-dimethyl- aminobenzaldehyde (0.4 g, 2.68 mmol, 1.35 equiv.) and piperidine (7mL), at 80 °C for 3h, following the general procedure, 0.52g (62%) of **B4** were obtained as an orange solid. ^{1}H NMR (300 MHz, DMSO-d_6) δ 7.99 (d, J = 3.2 Hz, 2H), 7.97 (d, J = 3.1 Hz, 1H), 7.78 (d, J = 8.8 Hz, 3H), 7.74 (d, J = 16.5 Hz, 1H), 7.64 (d, J = 9.1 Hz, 2H), 7.64 (d, J = 3.1 Hz, 1H), 7.19 (d, J = 16.5 Hz, 1H), 6.82 (d, J = 8.9 Hz, 2H), 4.10 (s, 6H), 3.05 (s, 6H). ^{13}C NMR (75 MHz, DMSO-d_6) δ 152.9, 149.7, 147.7, 132.6, 131.2, 126.6, 122.4, 113.2, 112.4, 101.4, 40.5, 33.6. HRMS, (ESI-TOF) m/z Calcd. for $C_{19}H_{22}N_3$ $[M]^+$: 292.1908, Found: 279.1805.

1.3.3. Synthesis of quadrupolar D-π-A+-A+-π-D bisbenzimidazoliums (Be2 and Be4).

Piperidine (2 equiv.) was added to a refluxing dry acetonitrile solution of 1,1',2,2',3,3'-hexamethyl-1H,3'H-5,5'-bisbenzimidazolium iodide ***9*** *(1 equiv.) and the corresponding arylaldehyde (2.4 equiv.), the mixture was refluxed at 80 - 85ºC for 6 - 2 hours. After completion of the reaction, the mixture was allowed to cool down at room temperature, then diethyl ether was added. The precipitate was collected by filtration and washed with diethyl ether. The solid residue was dried under vacuum to give* ***Be2*** *and* ***Be4****. Analytically pure sample was obtained by recrystallization from acetonitrile-ethanol.*

2,2'-bis [(E) - (4 - ((E) - (4-methoxyphenyl) ethenyl) phenyl) ethenyl] -1,1 ', 3,3'-tetramethyl-1H, 3'H-iodide -5,5'-bisbenzimidazolium iodide (Be2). From 1,1',2,2',3,3'-hexamethyl-1H,3'H-5,5'-bisbenzimidazolium iodide (103 mg, 0.18 mmol) 4-[(1E)-2-(4-methoxy phenyl)etenyl]-benzaldehyde (103 mg, 0.43 mmol, 2.4 equiv.), and piperidine (35 µL, 0.36 mmol, 2 equiv.), in dry acetonitrile (5mL), following the general procedure,

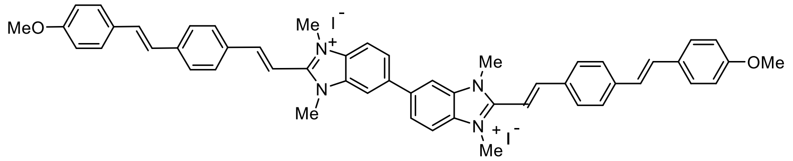

110 mg (60%) of **Be2** were obtained as an orange solid. IR, (KBr), ῡ$_{max}$ (cm^{-1}): 3020.4, 1626.7, 1589.0, 1507.0, 1250.5, 1174.9, 965.7, 826.2, 541.7. ^{1}H NMR (300 MHz, DMSO-d_6) δ (ppm): 8.55 (s, 2H), 8.27 – 8.18 (m, 4H), 7.96 (d, *J* = 8.2 Hz, 4H), 7.88 (d, *J* = 16.6 Hz, 2H), 7.75 (d, *J* = 8.1 Hz, 4H), 7.59 (d, *J* = 9.0 Hz, 6H), 7.40 (d, *J* = 16.4 Hz, 2H), 7.19 (d, *J* = 16.4 Hz, 2H), 6.98 (d, *J* = 8.3 Hz, 4H), 4.26 (s, 6H), 4.21 (s, 6H), 3.78 (s, 6H). MS (ESI^{+}) *m/z* (relative intensity, %): 380.29 [M]$^{2+}$ (100). HRMS, (ESI-TOF) m/z Calcd. For $C_{52}H_{48}N_4$ O_2[M]$^{2+}$: 380.4816, Found: 380.4821.

2,2' bis[(4-dimethylphenyl) ethenyl)] -1,1 ', 3,3'-tetramethyl-1H, 3'H-5, 5'-bisbenzimidazolium iodide (Be4). From 1,1',2,2',3,3'-hexamethyl-1H,3'H-5,5'-bisbenzimidazolium iodide (200 mg, 0.34 mmol) p-dimethyl-aminobenzaldehyde (124, mg, 0.84 mmol, 2.4 equiv.), and piperidine (69 μL, 0.69 mmol, 2 equiv.), in acetonitrile (5mL), following the general procedure, 176 mg (69%) of **Be4** were obtained as a light red solid. ^{1}H NMR (500 MHz, DMSO-d_6) δ (ppm): 8.47 (s, 2H), 8.18 (d, *J* = 8.7 Hz, 2H), 8.12 (d, *J* = 8.4 Hz, 2H), 7.84 – 7.76 (m, 6H), 7.19 (d, *J* = 16.4 Hz, 2H), 6.83 (d, *J* = 8.4 Hz, 4H), 4.22 (s, 6H), 4.15 (s, 6H), 3.06 (s, 12H). ^{13}C NMR (75 MHz, DMSO) δ (ppm): 152.4, 151.4, 150.6, 149.7, 147.2, 146.1, 137.5, 137.0, 132.9, 132.6, 131.9, 131.7, 130.8, 130.3, 125.8, 125.3, 121.7, 121.0, 113.8, 113.1, 111.9, 111.7, 110.7, 102.1, 100.6, 33.1, 33.0, 32.5. RMS, (ESI-TOF) m/z Calcd. for $C_{38}H_{42}N_6$ [M]$^{2+}$: 291.1730, Found: 291.1733.

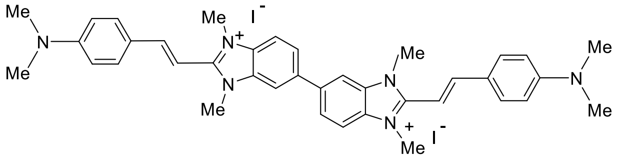

1.4 ^{1}H and ^{13}C NMR for all new compounds reported

^{1}H-NMR 500 MHz, DMSO-d_6

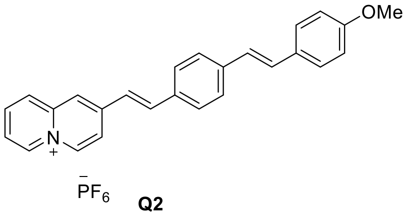

Q2

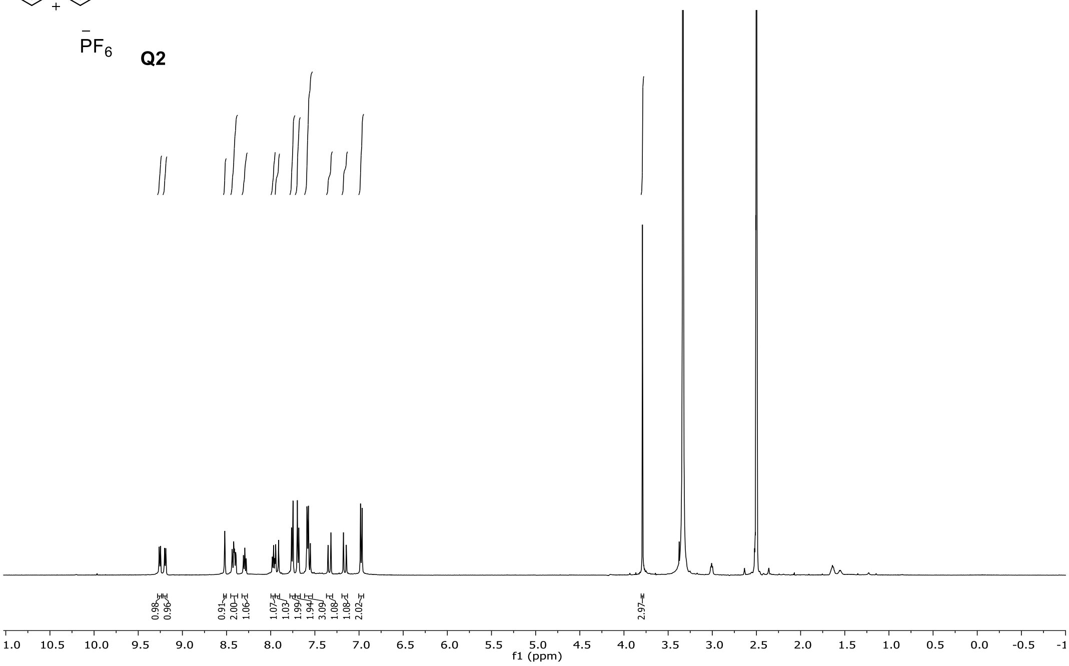

^{13}C-NMR 75 MHz, DMSO-d_6

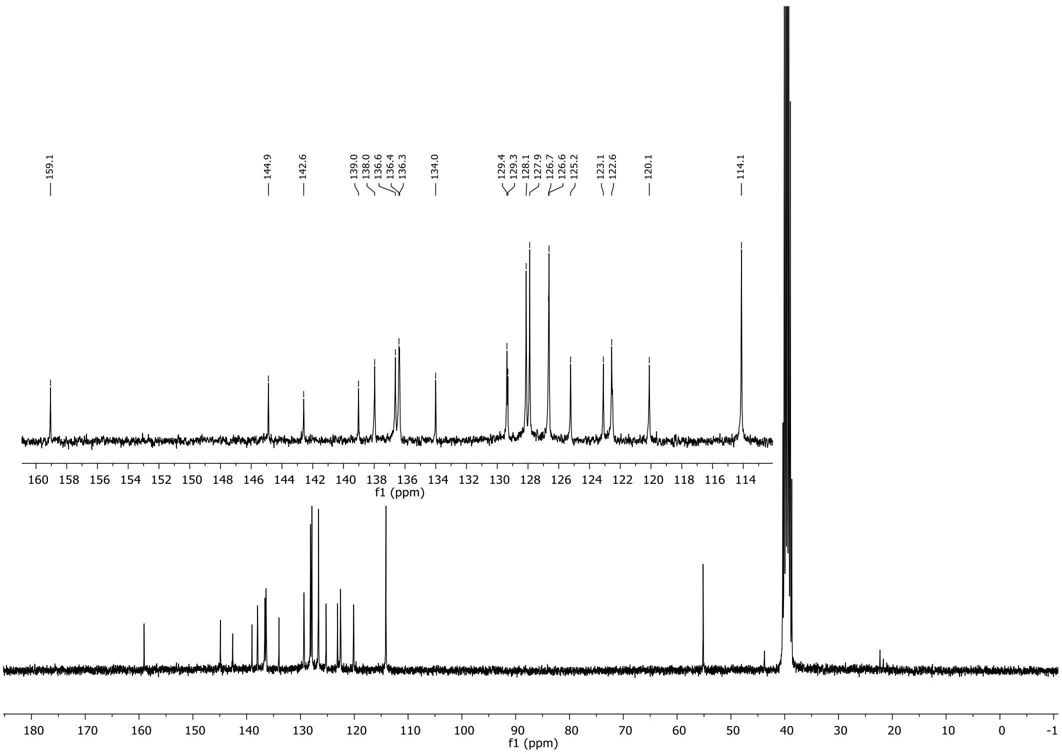

Figure S1. ^{1}H and ^{13}C NMR of **Q2**.

^{1}H-NMR 300 MHz, DMSO-d_6

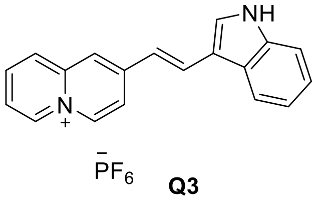

Q3

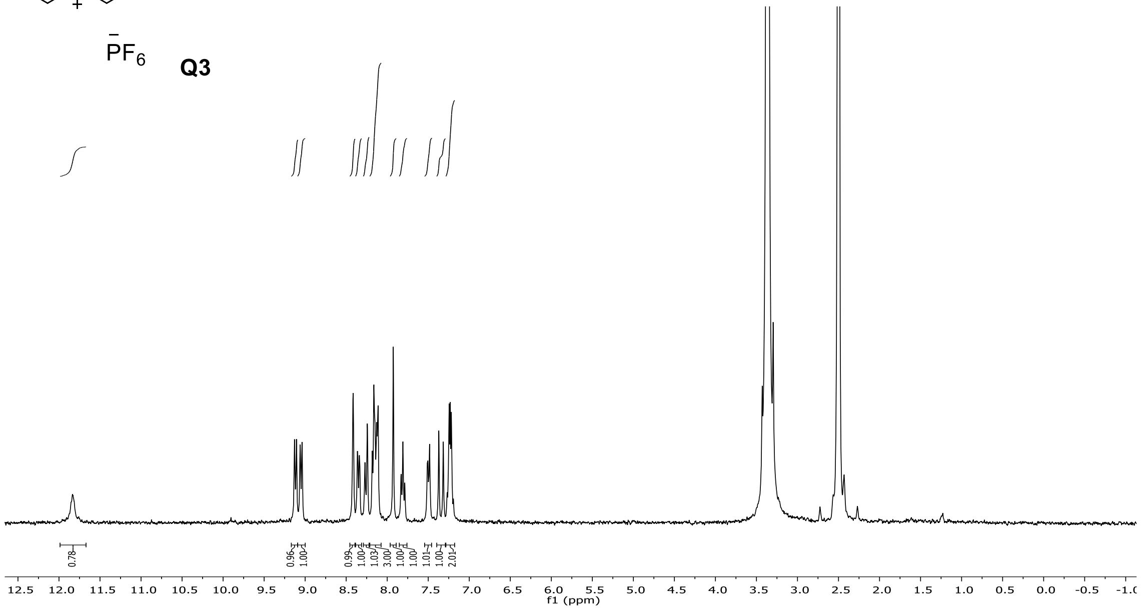

^{13}C-NMR 75 MHz, DMSO-d_6

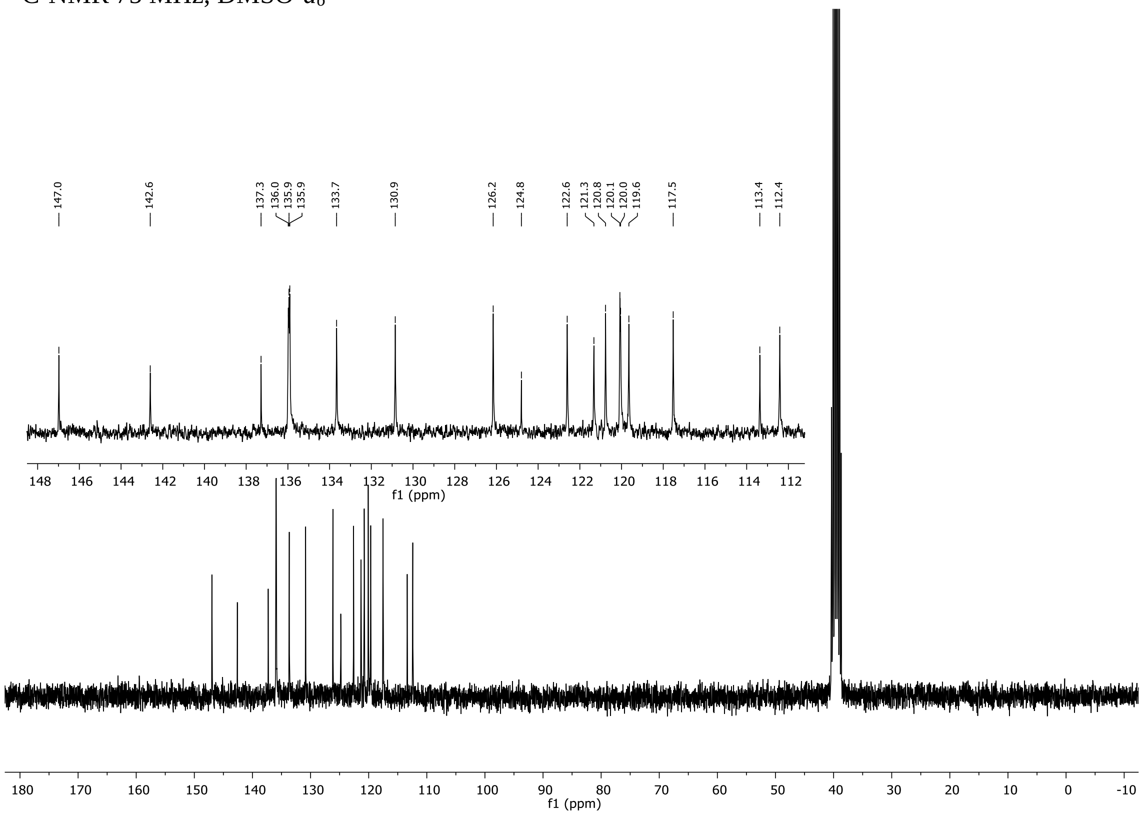

Figure S2. ^{1}H and ^{13}C NMR of **Q3**.

^{1}H-NMR 500 MHz, DMSO-d_6

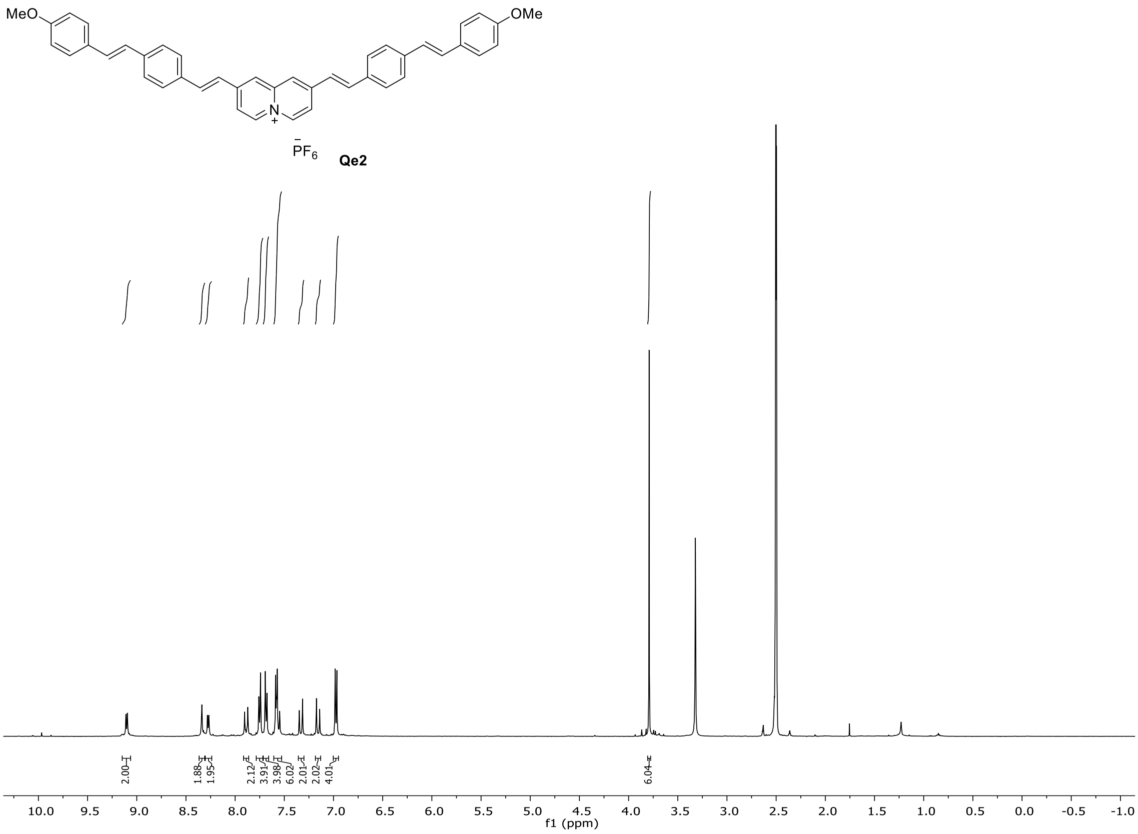

^{1}H-NMR 500 MHz, DMSO-d_6

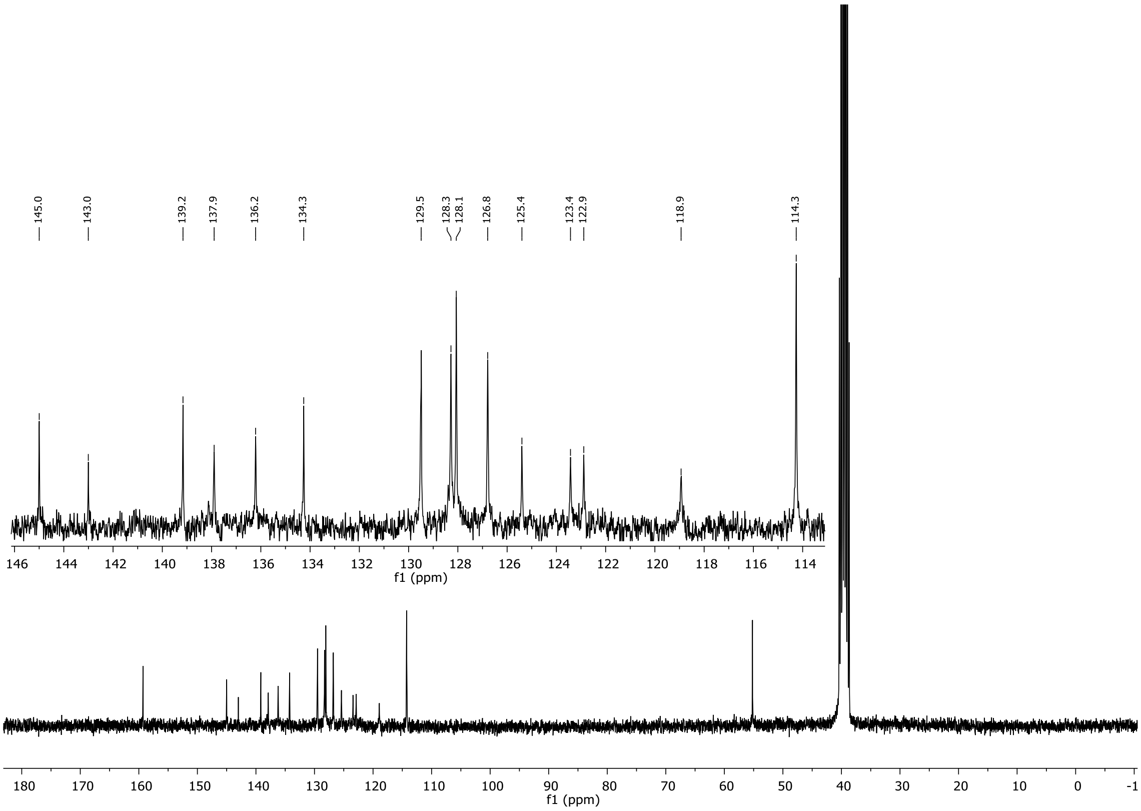

Figure S3. ^{1}H and ^{13}C NMR of **Qe2**.

^{1}H-NMR 500 MHz, DMSO-d_6

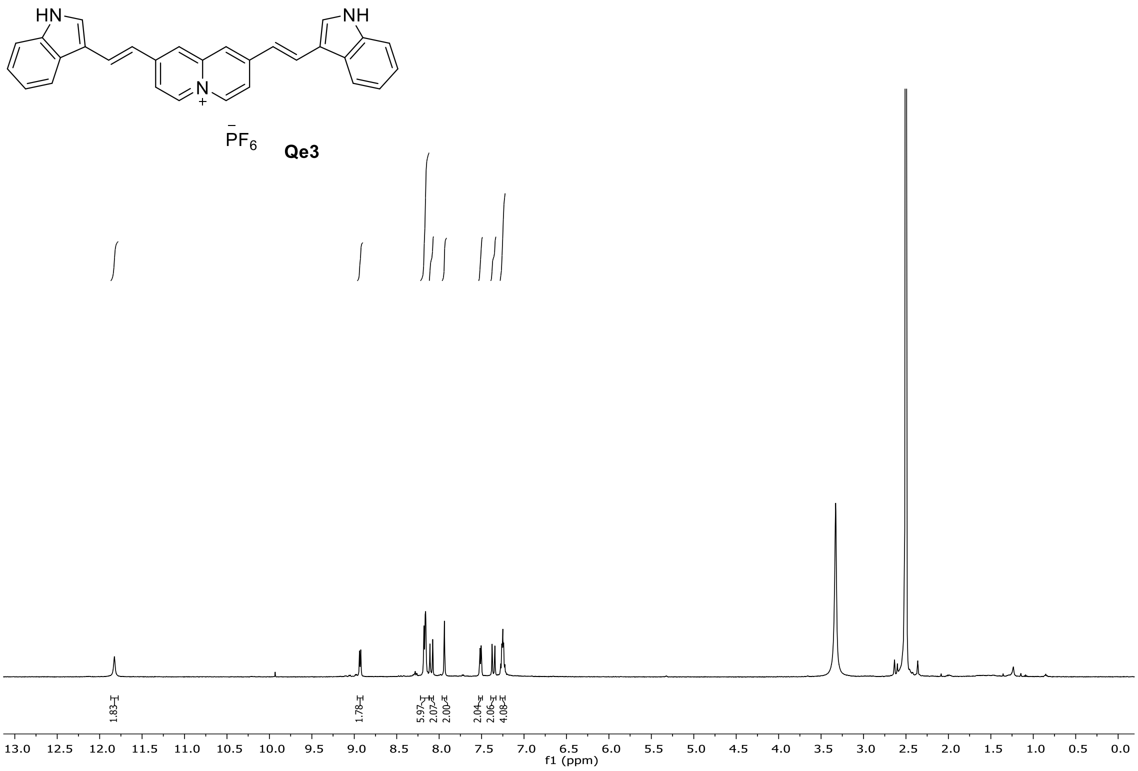

^{13}C-NMR 75 MHz, DMSO-d_6

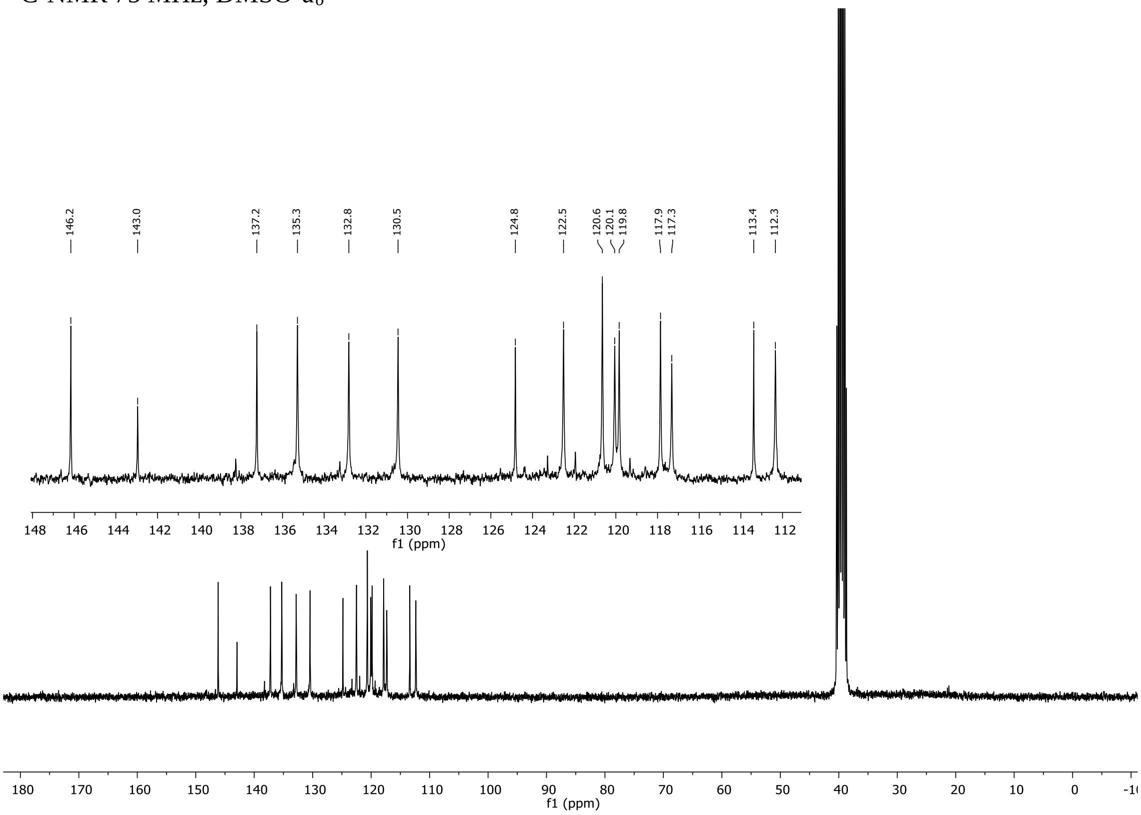

Figure S4. ^{1}H and ^{13}C NMR of **Qe3**.

^{1}H-NMR 500 MHz, DMSO-d_6

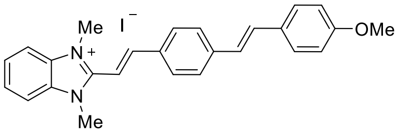

B2

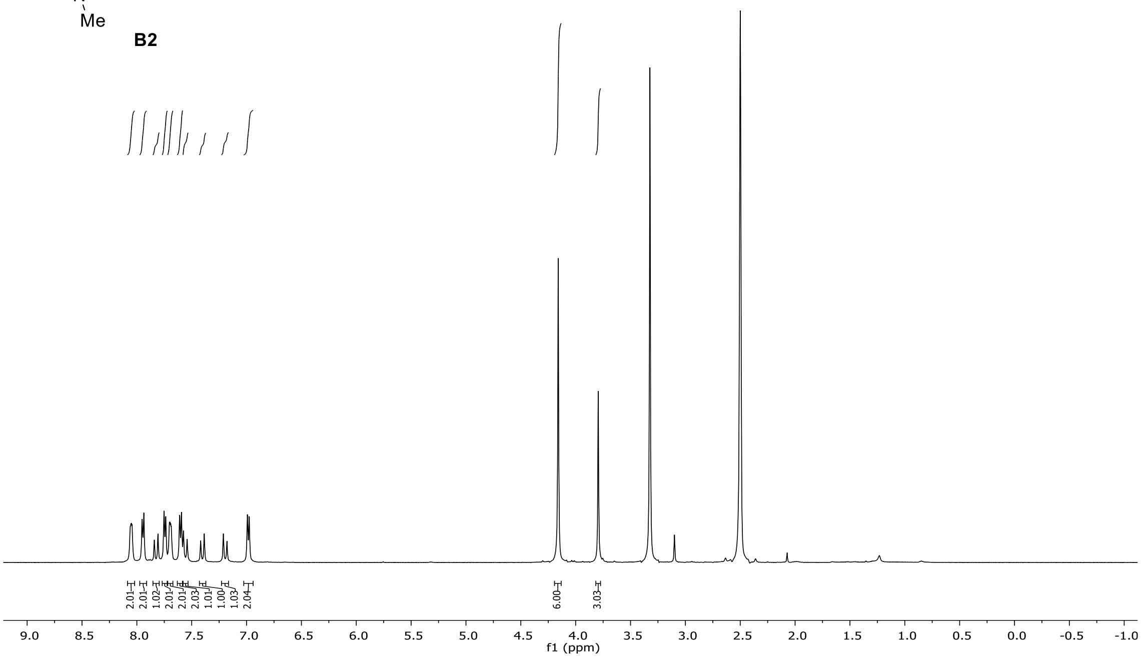

^{13}C-NMR 75 MHz, DMSO-d_6

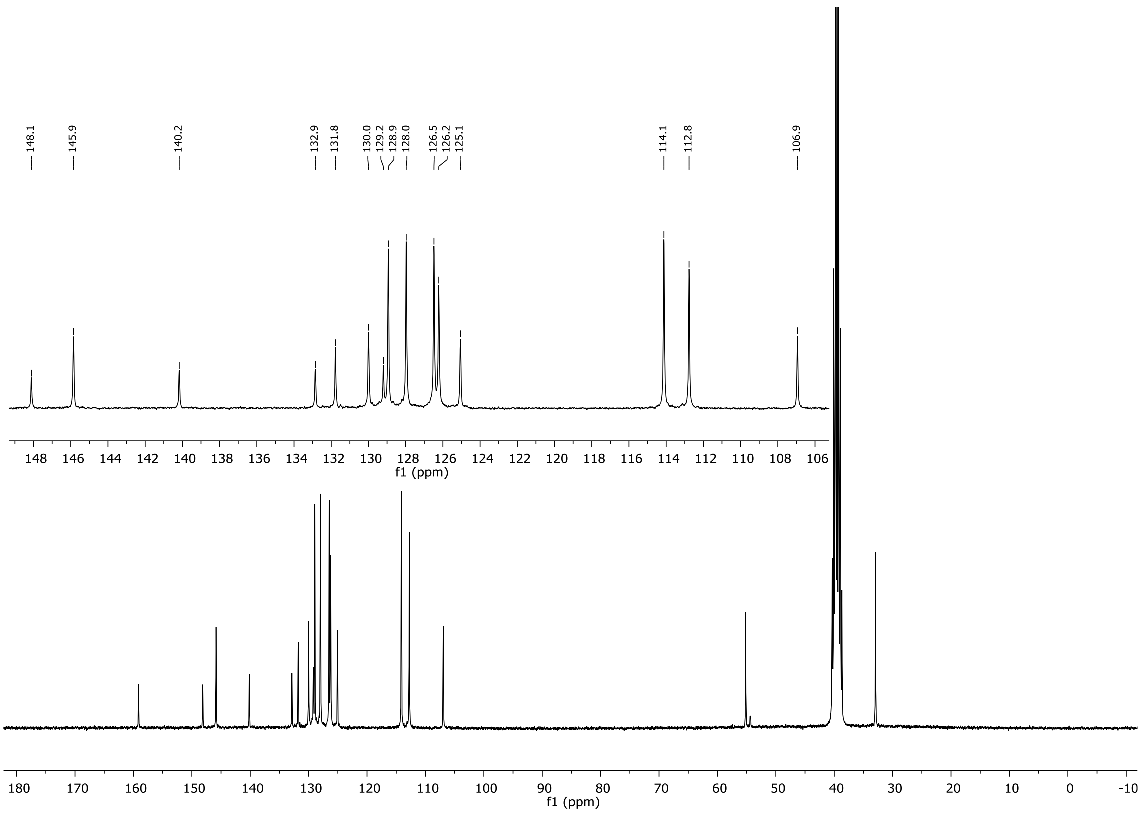

Figure S5. ^{1}H and ^{13}C NMR of **B2**.

^{1}H-NMR 300 MHz, DMSO-d_6

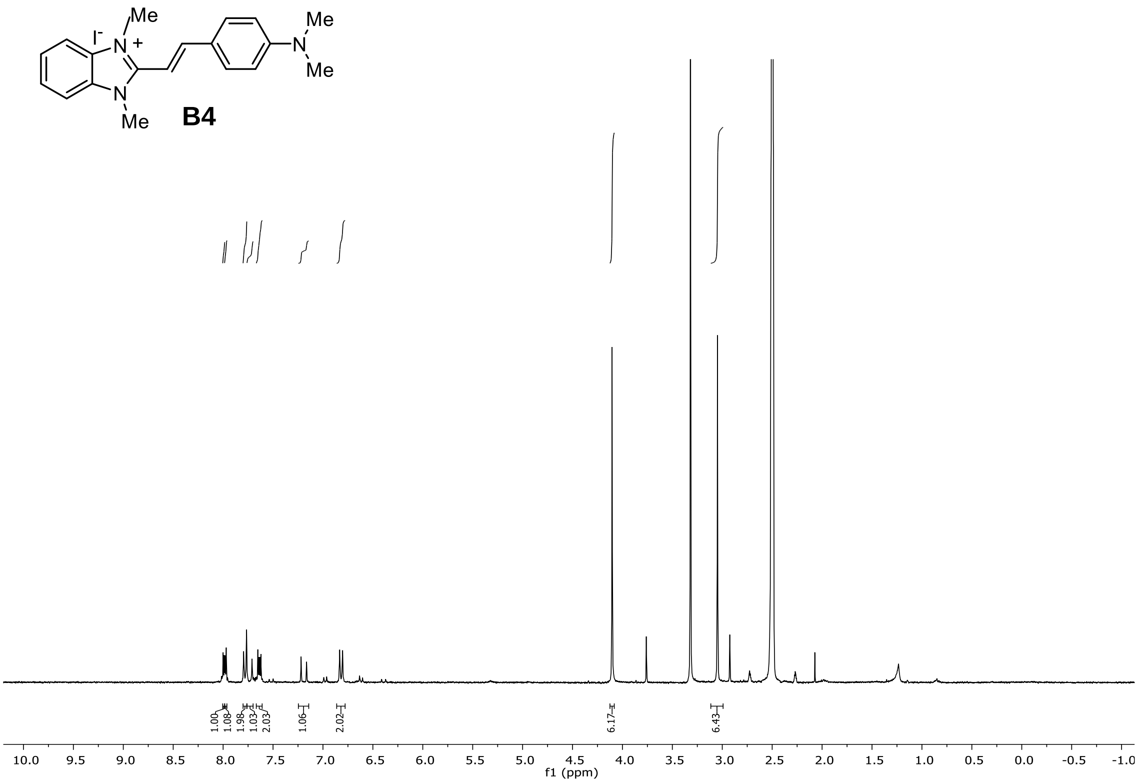

^{13}C-NMR 75 MHz, DMSO-d_6

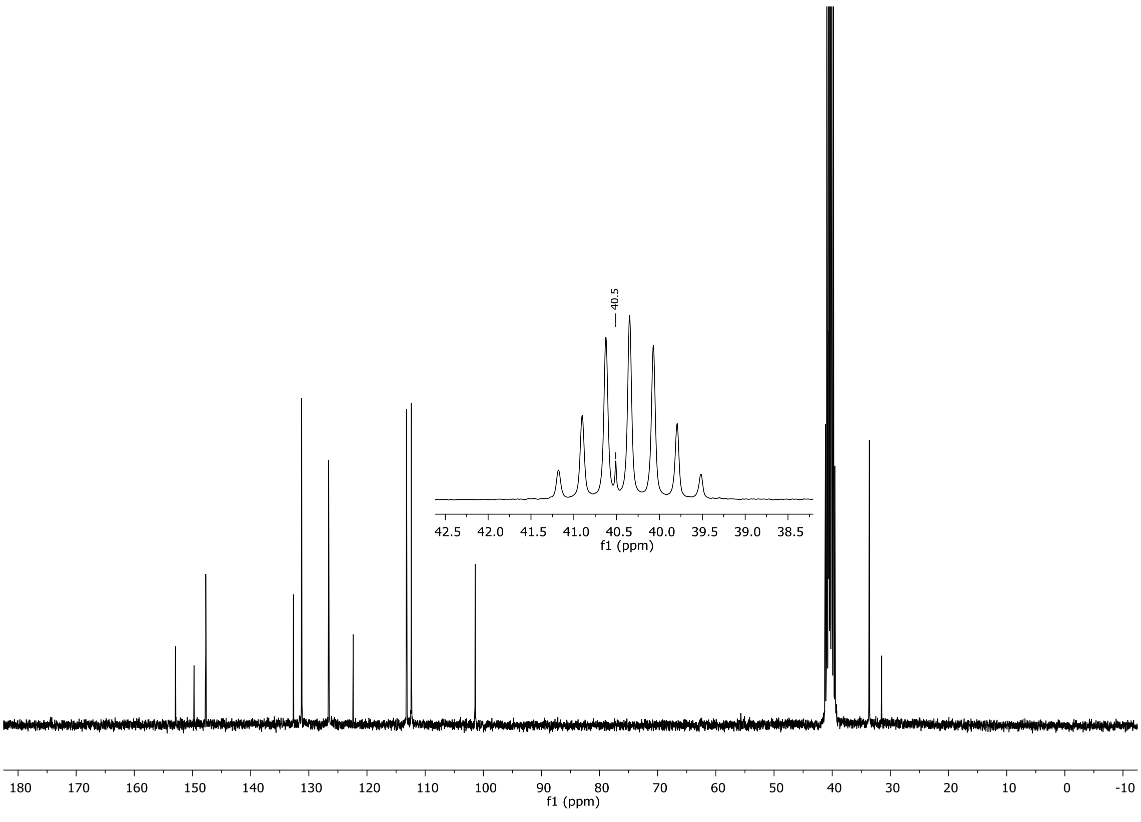

Figure S6. ^{1}H and ^{13}C NMR of **B4**.

^{1}H-NMR 300 MHz, DMSO-d_6

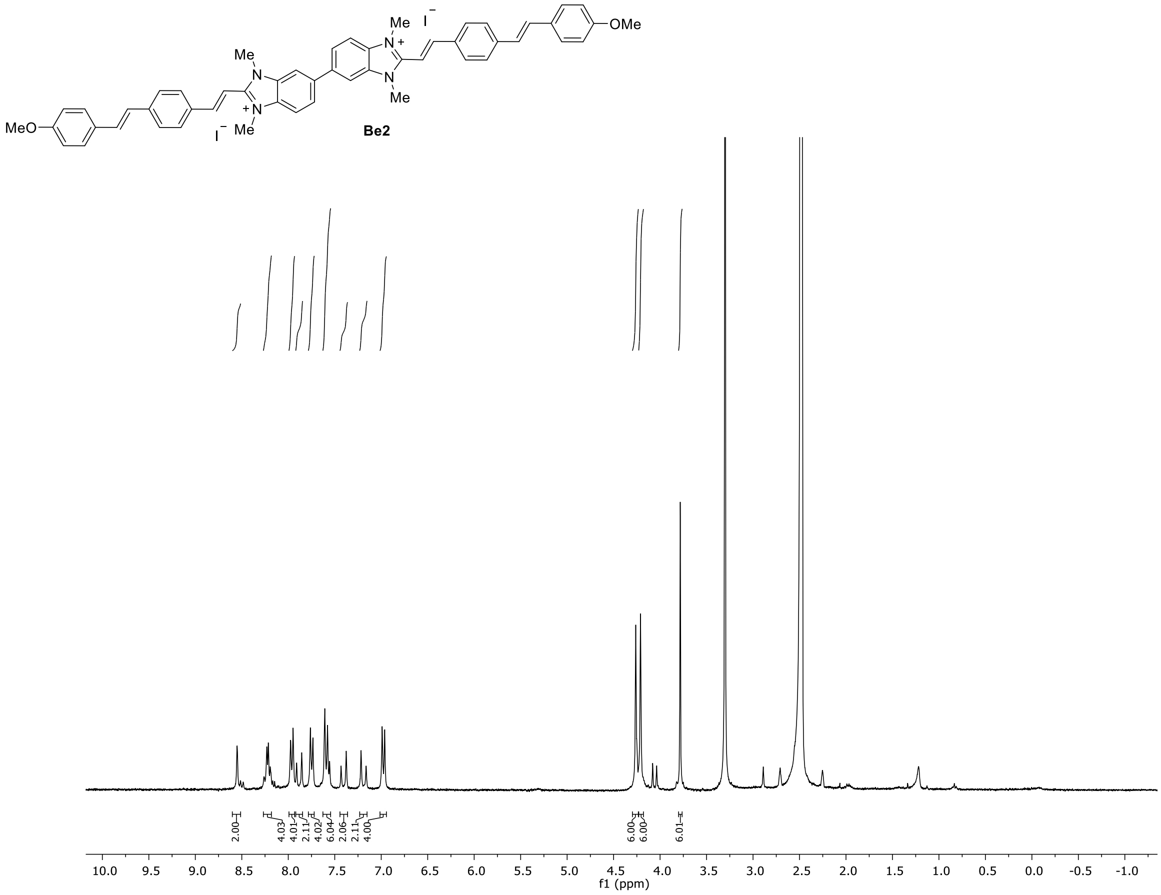

Figure S7. ^{1}H -NMR of **Be2**. ^{13}C-NMR not shown for **Be2** due to its reduced solubility in DMSO.

^{1}H-NMR 500 MHz, DMSO-d_6

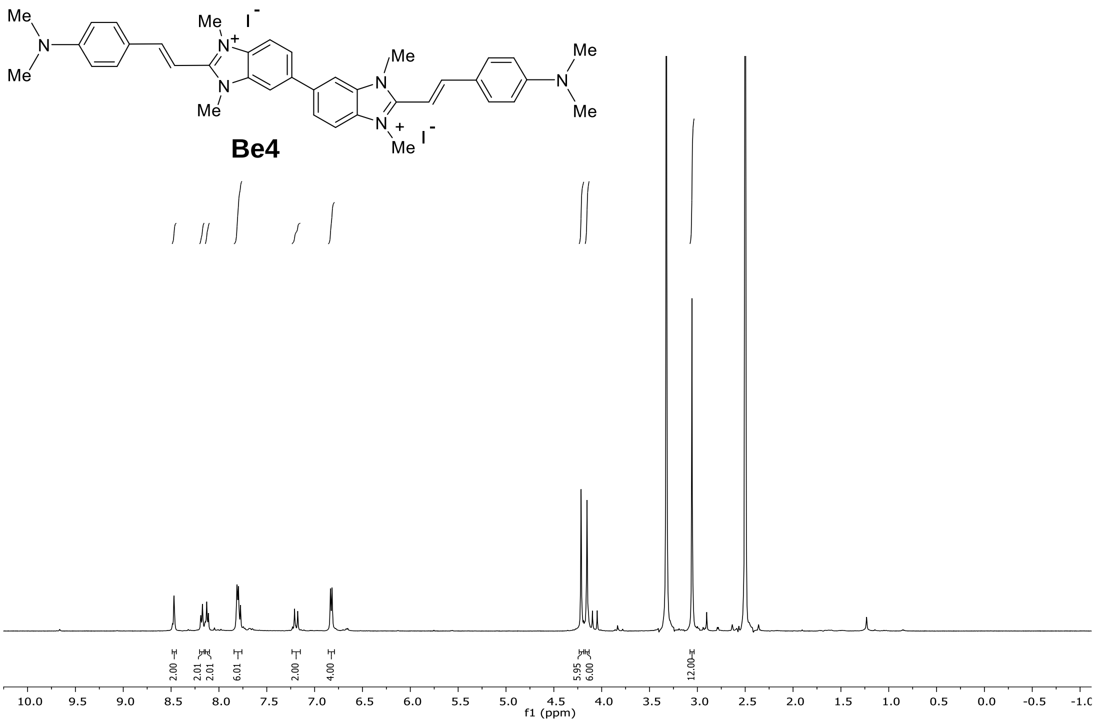

^{13}C-NMR 75 MHz, DMSO-d_6

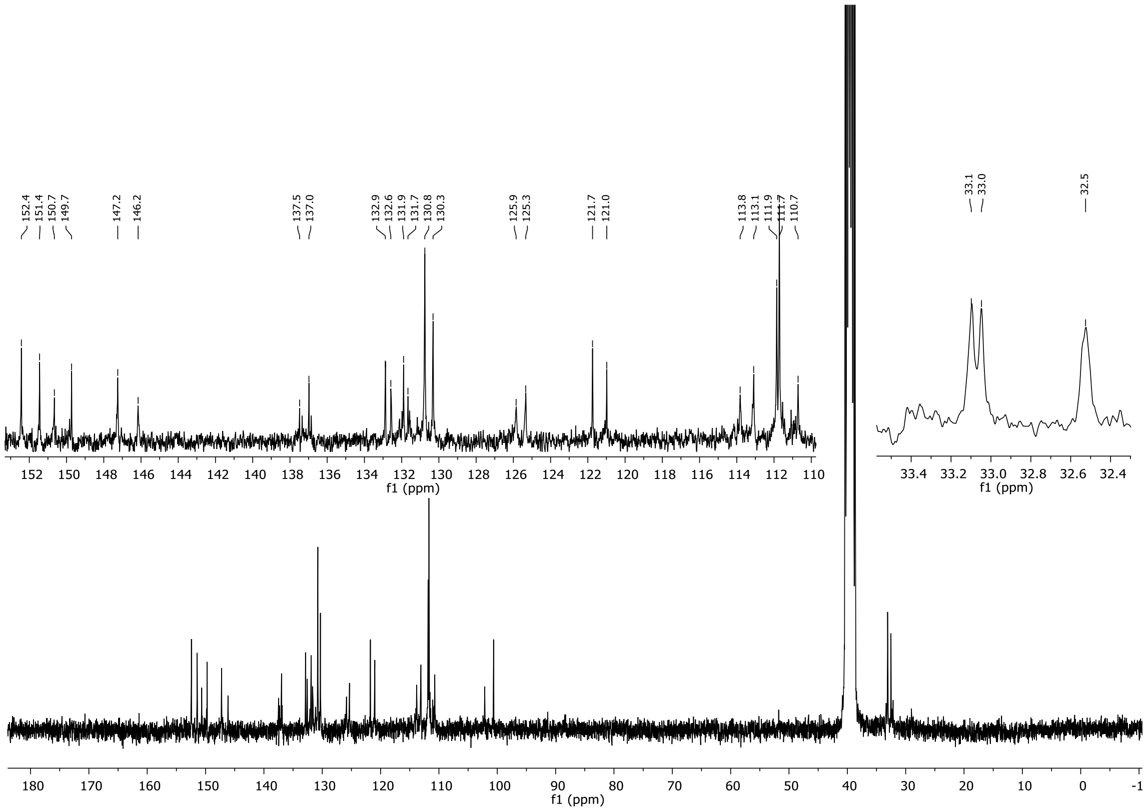

Figure S8. 1H and 13C NMR of **Be4**.

2. Optical properties

Table S1 Properties of the standards used to determine the quantum yield of the new compounds.

Compound	Standard	λexc (nm)	λem (nm)	ϕ	reference
Q2, B2 and Be2	Fluorescein in water at pH = 11	470-490	490 – 600	0.92	[10]
Q3, Qe3	Fluorescein in ethanol	470-490	490 – 600	0.79	[11]
Qe2, B4 and Be4	Rhodamine 101 in methanol	500-550	600 – 700	1.0	[10]

A summary of the optical properties of quinolizinium and benzimidazolium derivatives in DMSO is presented in Table S2 together with the calculated wavelength of the lowest energy transition and the percentage contribution of the most important excitation to the configuration interaction (CI) expansion. Calculated data were obtained from Time Dependent Density Functional Theory (TD-DFT) calculations on the optimized geometry of the cation in DMSO using the hybrid exchange-correlation density functional CAM-B3LYP and the 6-311+G(d,p) basis function. Vibrational calculations were performed to ensure that a global minimum, with no imaginary frequencies. Solvent contribution was accounted for using the polarizable conductor-like polarizable continuum model (CPCM). All the calculations were performed on Gaussian 09.[12]

Table S2. Optical properties of the quinolizinium and benzimidazolium derivatives in DMSO: maximum linear and nonlinear (two-photon) absorption and emission wavelength (λ_{max}OPA, λ_{max}TPA and λ_{max}OPE), Stokes shift ($\Delta\nu_{ST}$), fluorescence quantum yield (ϕ), maximum one- and two-photon absorption cross-section (σ_1 and σ_2), and maximum two-photon brightness ($\sigma_2\phi$), calculated wavelength for the lowest energy transition (λ_{max}OPA calc) and corresponding configuration interaction contribution CI (%).

Comp [a]	λ_{max}OPA (nm)	λ_{max}OPE (nm)	$\Delta\nu_{ST}$ (cm^{-1})	ϕ	$\sigma_1 \times 10^{-16}$ (cm^2)	λ_{max}TPA (nm)	σ_{2max} (GM)	$\sigma_2\phi$ (GM)	λ_{max}OPA calc (nm)	CI (%)
**Q2**	422	688	9162	0.24	1.99	890	266	65	407	H → L (76%)
**Q3**	443	566	4906	0.10	1.38	890	92	10	398	H → L (92%)
**Qe2**	465	701	7240	0.003	1.60	820	1253	4	433	H → L (64%)
**Qe3**	488	603	3908	0.07	2.06	850	482	34	427	H → L (85%)
**Qe3´**	490	601	3769	0.07	0.11	850	25	2		
**B2**	380	628	10392	0.34	1.49	810	55	19	379	H → L (82%)
**B4**	400	552	6884	0.01	0.84	810	75	1	382	H → L (93%)
**Be2**	407	635	8822	0.17	2.94	830	189	32	515	H → L (44%)<br>H-1 → L+1 (34%)
**Be4**	448	562	4528	0.01	2.48	860	281	4	395	H → L (53%)<br>H-1 → L+1 (38%)

[a] The counterion is $PF6^-$ and I^- in the quinolizinium and benzimidazolium series. The prime in **Qe3** indicates that the BF_4^- anion was used instead of the PF_6^- anion.

For the quinolizinium cation **Qe3** we studied the effect of the counterion anion by replacing the hexafluorophosphate counterion ($PF6^-$) for a more hydrophilic tetrafluoroborate anion ($BF4^-$) in **Qe3'**. The driving force for this study was to improve the water solubility of the compounds. The exchange of the counterion anion did not affect the shape of the absorption and emission spectra nor the quantum yields, but it decreased significantly the one- and two-photon absorption cross-sections. On accounts of this effect, both linear and nonlinear brightness of **Qe3** decreased by more than one order of magnitude. A systematic evaluation of the anion exchange effect is beyond the scope of this work. Nevertheless, we noticed that the anion exchange effect appears to be stronger in compounds with a weaker push-pull effect. For the quinolizinium derivatives with a strong dimethylamine electron donor (VDMA in ref. 4) no anion exchange effect was observed in the absorption cross-sections, whereas in the equivalent compound with a weaker methoxy electron donor (VMOP in ref. 4) the exchange of $PF6^-$ by $BF4^-$ resulted in a decrease of the cross-section by a factor of 2, and in the analogous but more extended **Qe3** the drop was larger than one order of magnitude. Thus, to avoid effects related with the nature of the counterion we kept the counterion constant within each series (quinoliziniums with $PF6^-$ and benzimidazoliums with I^-). Due to the strong electron donor nature of the dimethylamine donor in the benzimidazolium series no strong counterion effect is expected.

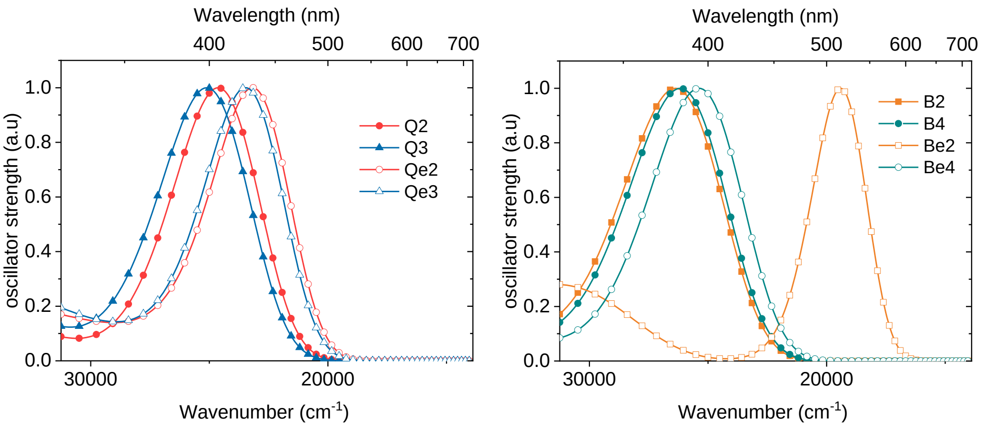

Figure S9. Calculated normalized absorption spectra based on the singlet-singlet transitions predicted in the 300-700 nm range. The spectra were simulated as a sum of gaussian functions centered at the calculated transition energies with integrated intensities corresponding to the calculated oscillator strength and full-width at half maximum of 30 nm.

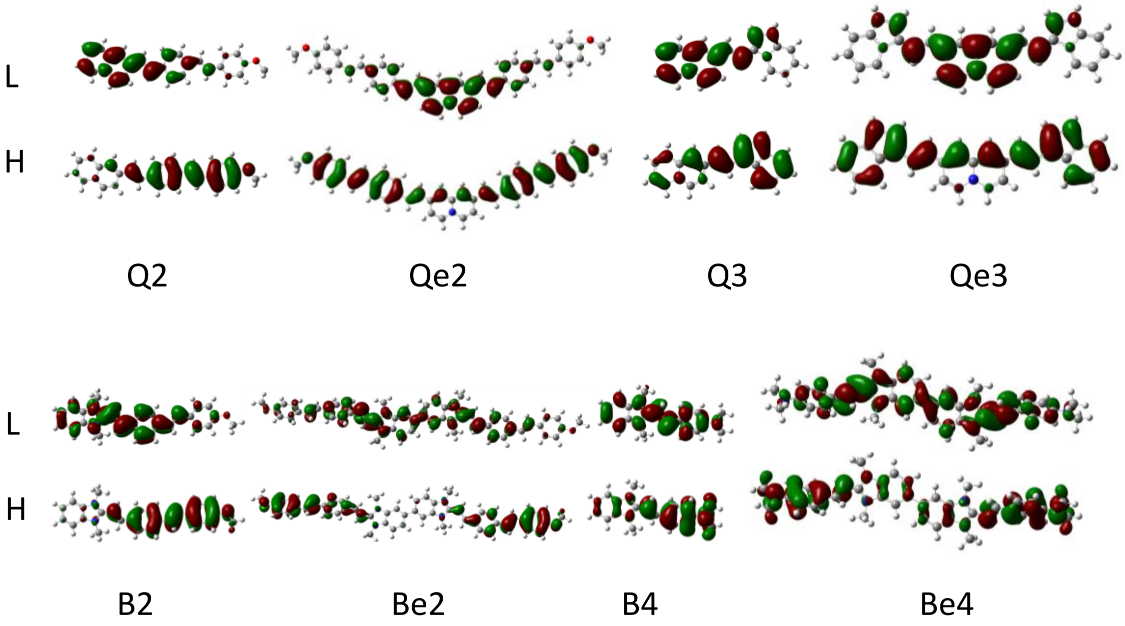

Figure S10. Isodensity surface of the frontier molecular orbitals involved in the strongest and lowest energy transition in the 300-700 nm region.

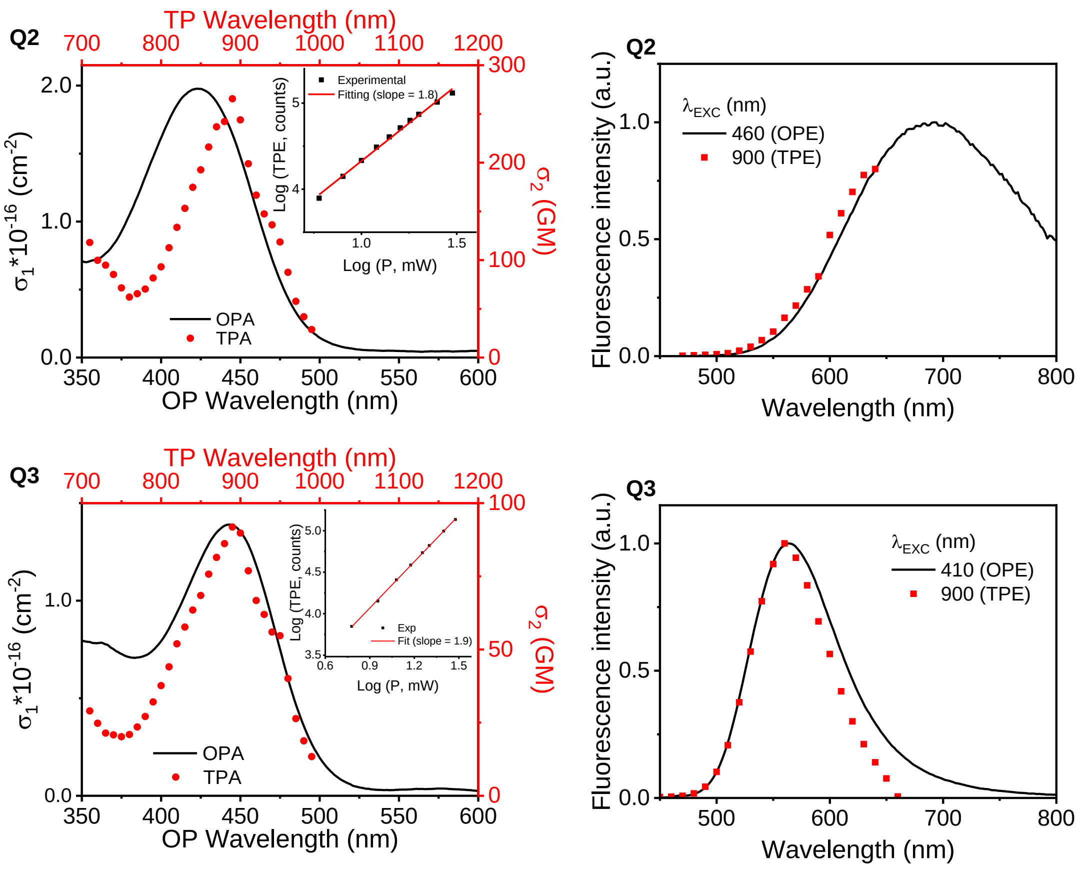

Figure S11. Absorption (left) and emission (right) spectra of the dipolar quinolizinium derivatives (**Q2** and **Q3**) in DMSO. The TPA spectra are shown using a wavelength scale that is twice the scale of the OPA spectra. The inserts on the left absorption spectra are the log-log plots of the photon counts as a function of the nonlinear excitation power showing a slope of ≈ 2 that confirms the quadratic dependence of the two-photon induced emission.

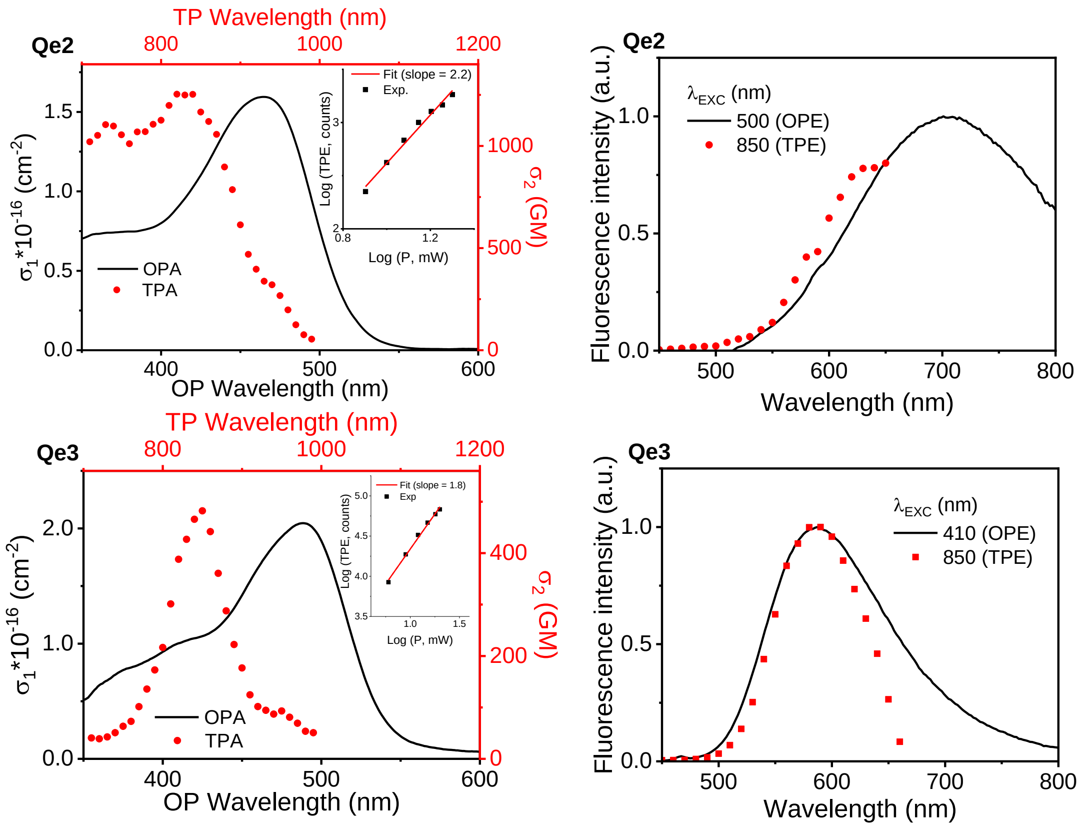

Figure S12. Absorption (left) and emission (right) spectra of the quadrupolar quinolizinium derivatives (**Qe2** and **Qe3**) in DMSO. The TPA spectra are shown using a wavelength scale that is twice the scale of the OPA spectra. The inserts on the left absorption spectra are the log-log plots of the photon counts as a function of the nonlinear excitation power sowing a slope of ≈ 2 that confirms the quadratic dependence of the two-photon induced emission. The blueshift of the two-photon absorption maxima with respect to the one-photon absorption maxima suggest that the energy levels involved in the lowest energy nonlinear transition are higher in energy with respect to the S_0->S_1 transition. This is a common observation in centrosymmetric molecules (**Be2** and **Be4**) and in nearly centrosymmetric structures such as **Qe1**, **Qe2** or **Qe3**.

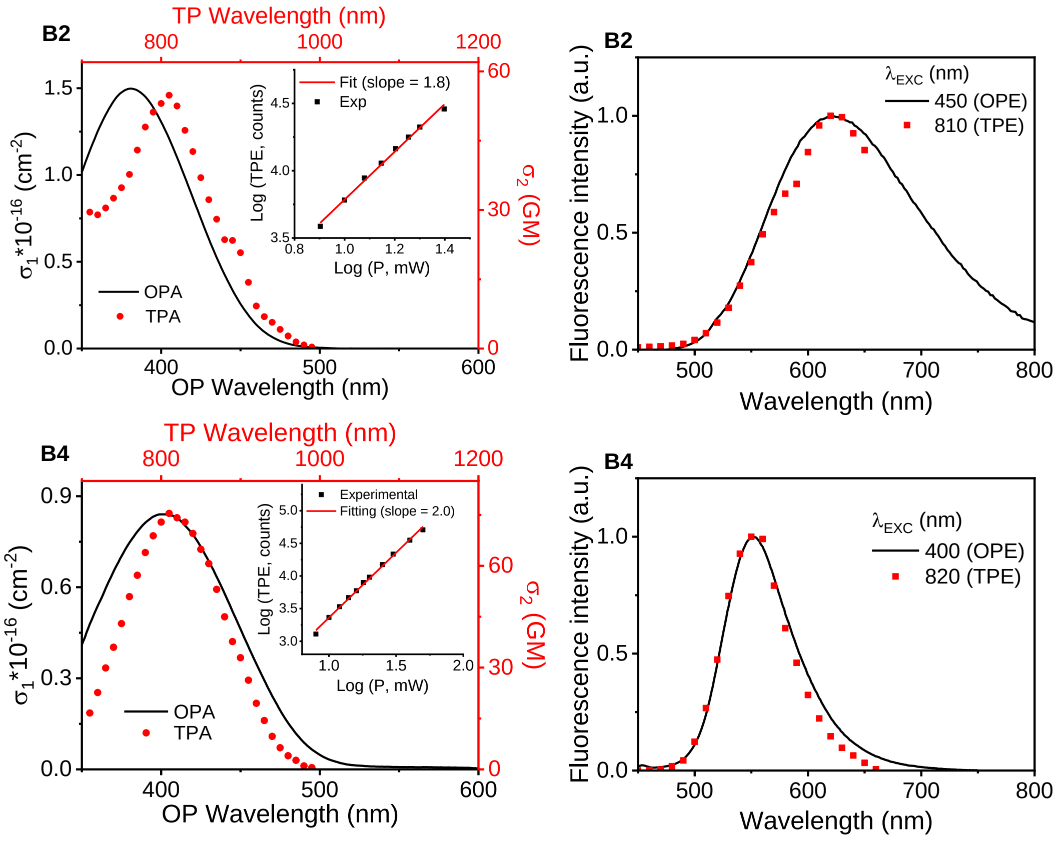

Figure S13. Absorption (left) and emission (right) spectra of the dipolar benzimidazolium derivatives (**B2** and **B4**) in DMSO. The TPA spectra are shown using a wavelength scale that is twice the scale of the OPA spectra. The inserts on the left absorption spectra are the log-log plots of the photon counts as a function of the nonlinear excitation power sowing a slope of ≈ 2 that confirms the quadratic dependence of the two-photon induced emission.

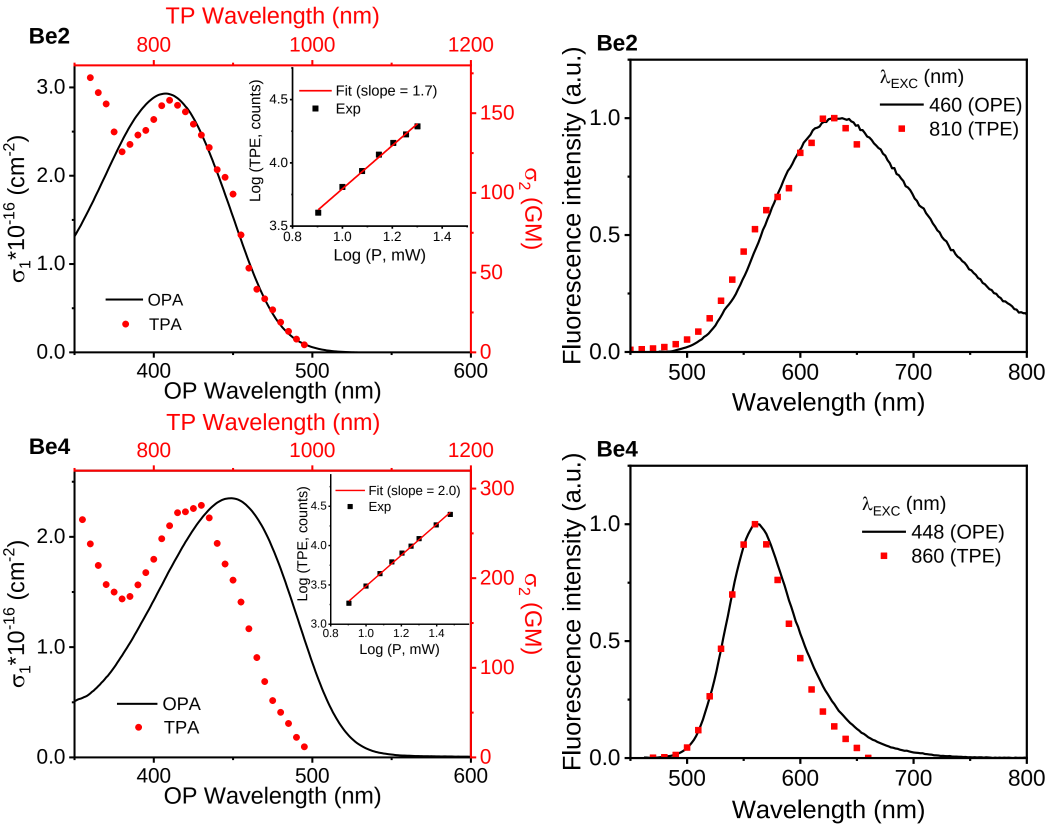

Figure S14. Absorption (left) and emission (right) spectra of the dipolar benzimidazolium derivatives (**Be2** and **Be4**) in DMSO. The TPA spectra are shown using a wavelength scale that is twice the scale of the OPA spectra. The inserts on the left absorption spectra are the log-log plots of the photon counts as a function of the nonlinear excitation power sowing a slope of ≈ 2 that confirms the quadratic dependence of the two-photon induced emission.

3. Cell culture and staining

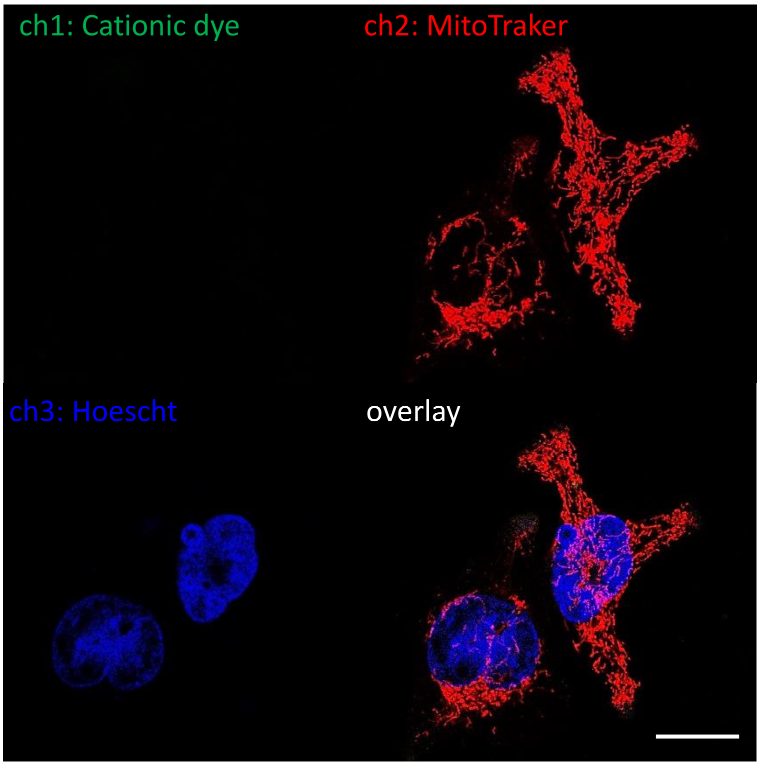

Figure S15. Control experiment showing the fluorescence confocal microscopy images of HEK 293T cells incubated with MitoTracker Red and Hoechst 33342, without any lipophilic cation. Channel 1 (ch1) shows the image collected using the typical acquisition parameters used to image the cationic dyes (green, λ_{exc} = 458 nm, λ_{em}= 500-600 nm), channel 2 shows the emission of MitoTracker Red (in red, λ_{exc} = 514 nm, λ_{em}= 600-700 nm) and channel 3 shows the emission of Hoechst 33342 (in blue, λ_{exc} = 780 nm, λ_{em}= 400-500 nm). The overlay of the isolated channels is shown in the bottom right panel. The lack of green emission in channel 1 confirms that emission of MitoTracker is well isolated in channel 2. Scale bar common in all images: 20 µm.

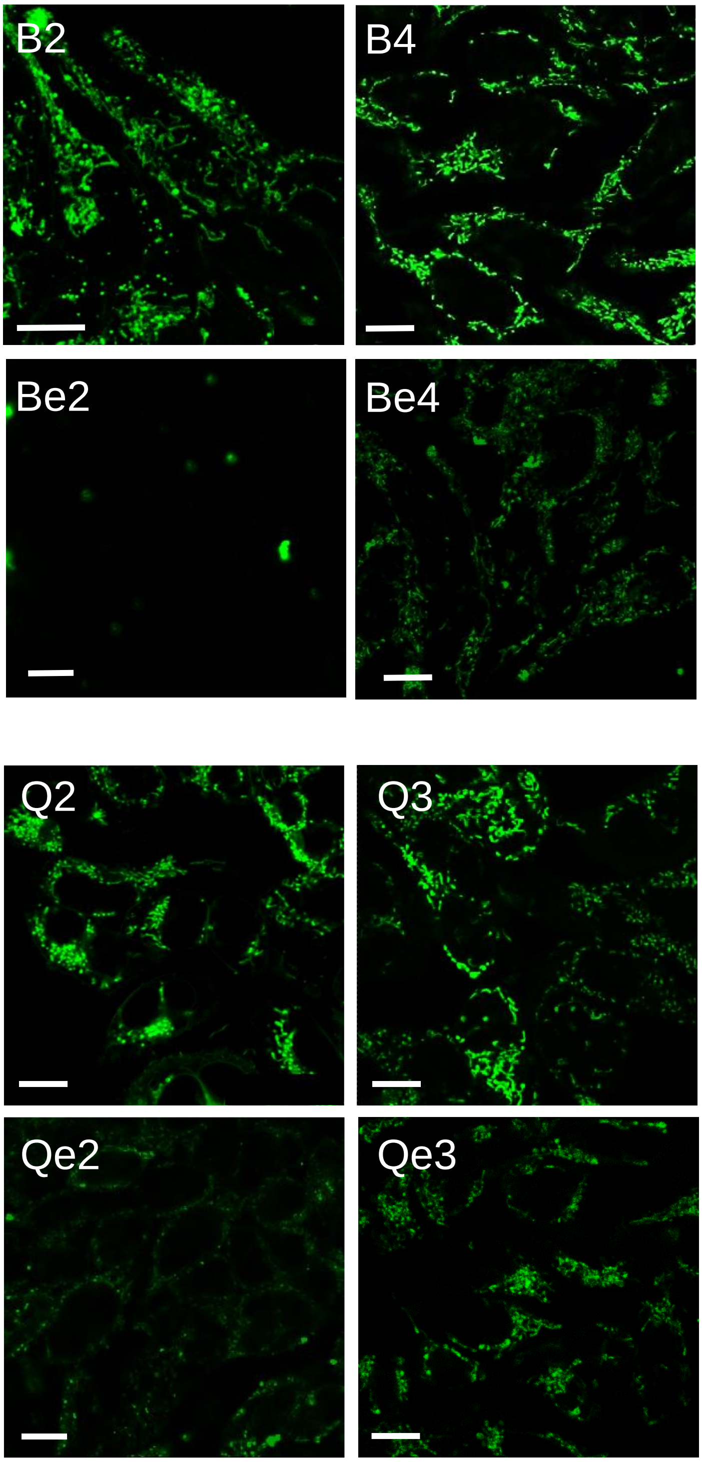

Figure S16. Fluorescence confocal microscopy image of all the quinolinium and benzimidazolium compounds incubated in HEK 293T cells with no other dye added. The emission was collected in the 500-600 nm range upon excitation at 458 nm. Scale bar equals to 10 μm. Similar excitation and emission conditions were used for all the compounds.

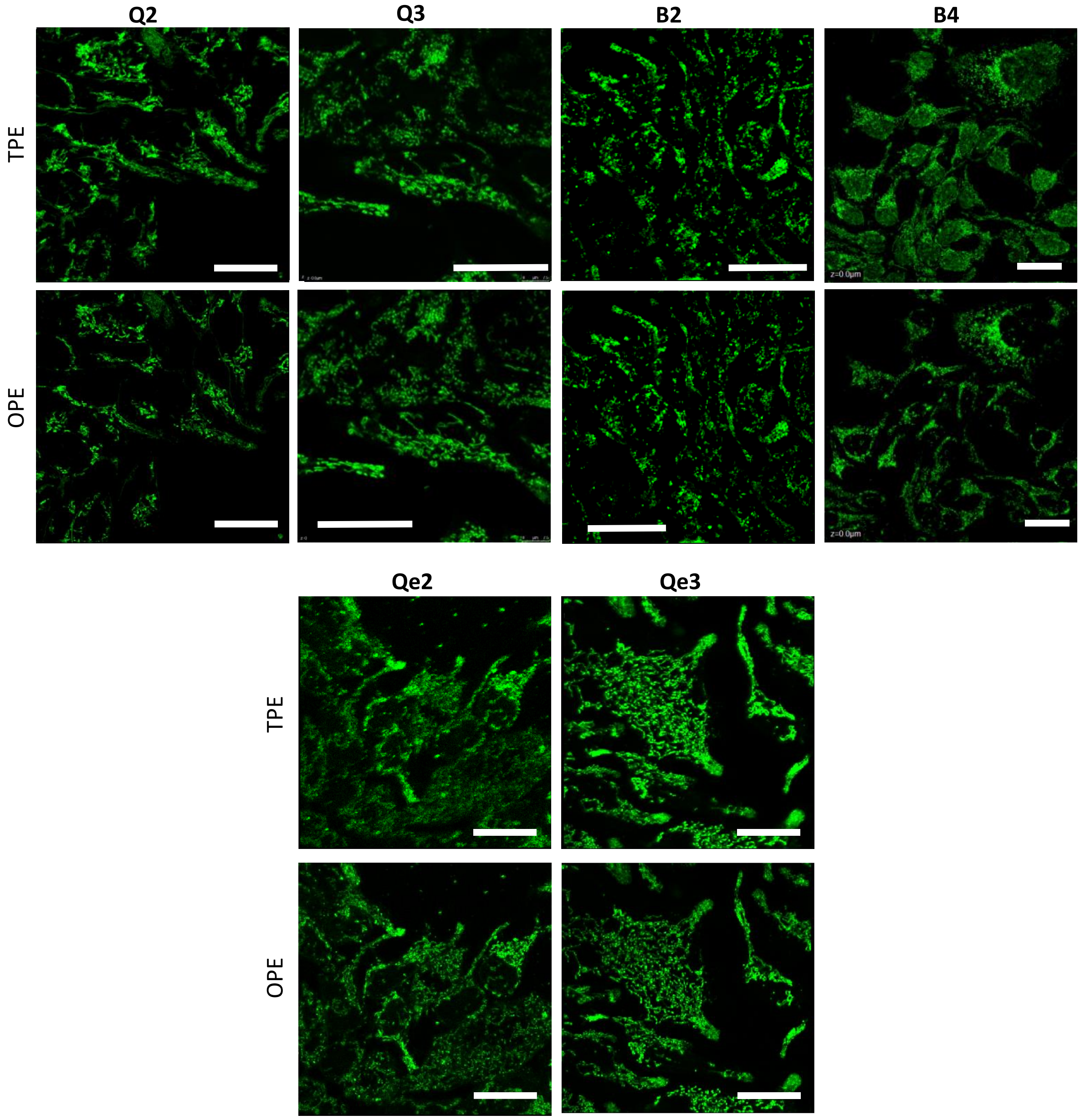

Figure S17 Fluorescence microscopy images showing HEK293T cells incubated with the cationic dyes under one-photon excitation (OPE) at 458 nm and two-photon excitation (TPE) at 800-840 nm with emission at 500-700 nm. Scale bar equals to 25 μm.

4. Photoirradiation effect

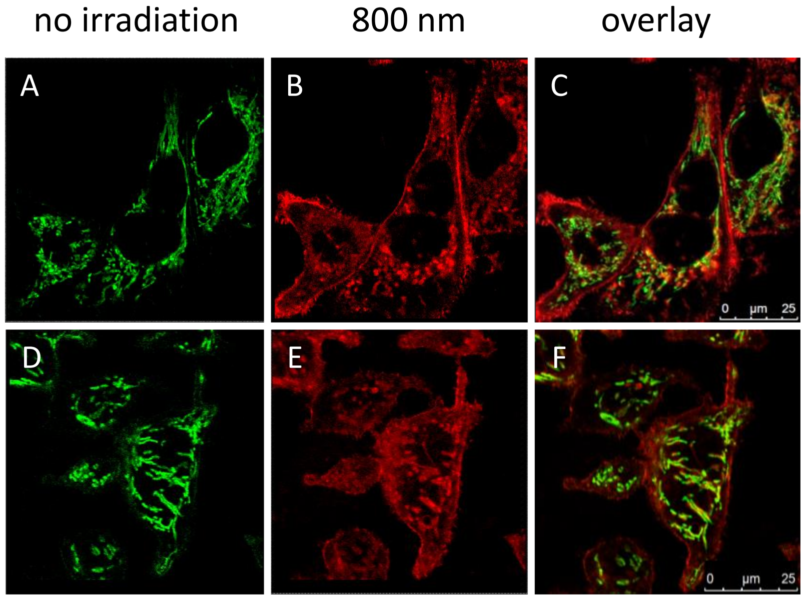

Figure S18 Fluorescence microscopy images showing the effect of two-photon excitation (800 nm) of HEK293T cells incubated with 1.8 µM of **Q2**. Top panels (A-C) and bottom panels (D-F) correspond to irradiation for 5 min with 8 mW and 22 s with 16 mW, respectively. The light was focused through a 63x 1.2 N.A. water immersion objective and the irradiation was done in scanning mode with 400 Hz per line collecting 512 x 512 pixel images. The emission of **Q2** was collected at 500-700 nm.

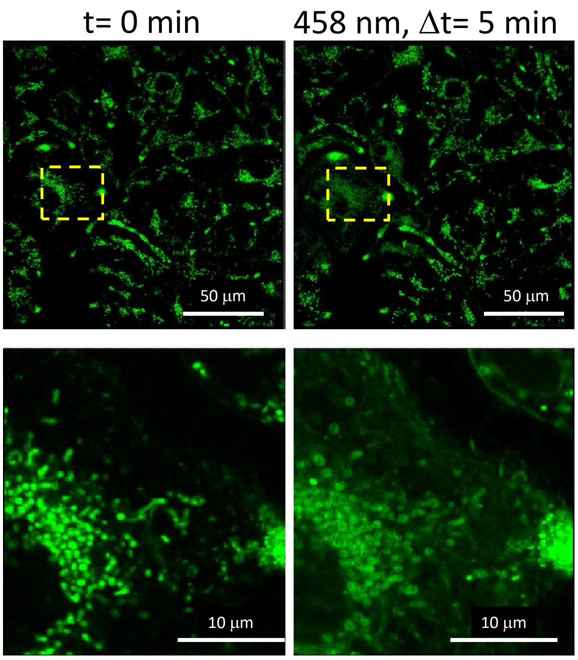

Figure S19. Fluorescence microscopy images showing the effect of one-photon excitation (458 nm, 20 µW, 5 min) of HEK293T cells incubated with 1.8 µM of **Q2**. The light was focused through a 63x 1.2 N.A. water immersion objective and the irradiation was done in scanning mode with 400 Hz per line collecting 512 x 512 pixel images. The emission of **Q2** was collected at 500-700 nm. Left panels (A and B) are images taken before irradiation. The right panels (C and D) where taken after irradiation. The lower panels are an amplification of the highlighted area in the top panels.

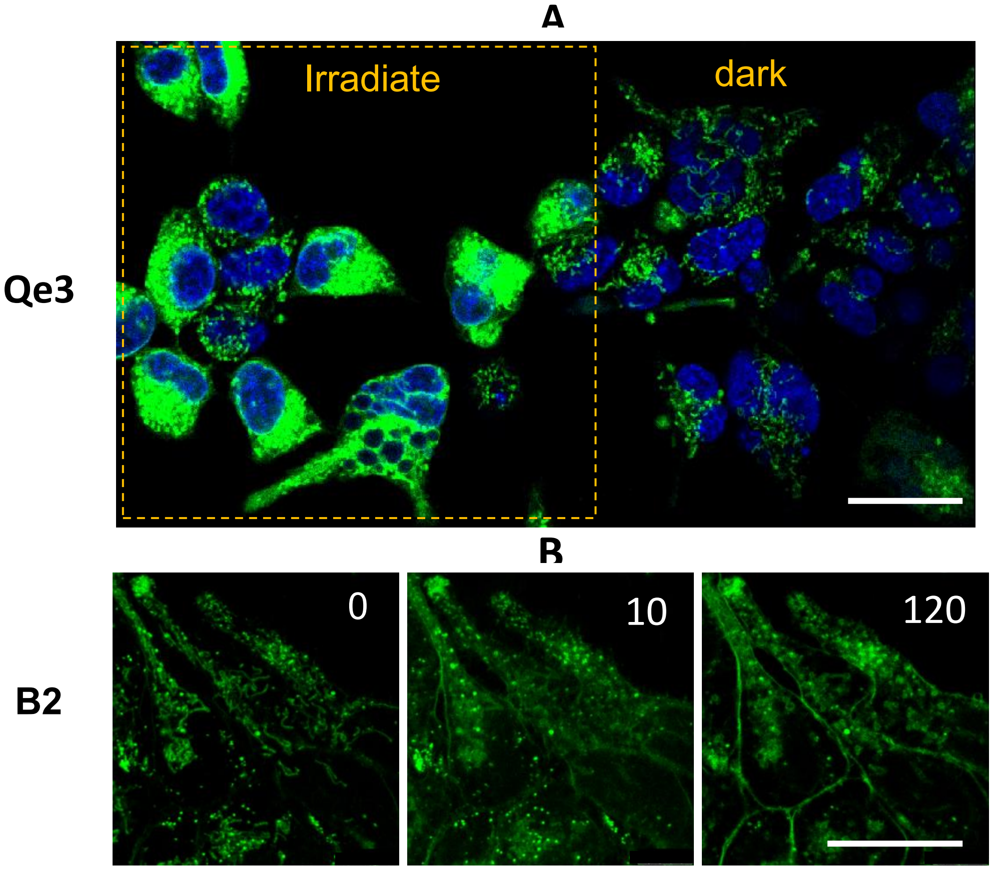

Figure S20 - Mitochondrial morphology changes in HEK293T cells incubated with 2.5 mM of **Qe3** (panel A) and **B2** (panel B) induced by UV irradiation with the CW lamp of the microscope. For **Qe3**, Panel A shows cell that were irradiated (left) vs cells kept in the dark (right), within the same field of view. The exposed cells show mitochondria swelling with vesicular shape and bleb formation, the emission intensity of **Qe3** increases upon irradiation. Unexposed cells show mitochondria with elongated shape. For **B2**, Panel B shows the effect of 10 s and 120 s of exposure. In this case mitochondria swelling and formation of vesicles is accompanied by a decrease in the intensity of **B2** upon exposure. To compensate for a decrease of intensity of a factor of 3 observed at 120 s the image intensity was adjusted to better illustrated the photoinduced changes. The emission of **B2** and **Qe3** were collected in the 500-600 nm region. Scale bar corresponds to 25 μm.

Movie with visible excitation effect:
https://youtu.be/K9jV0THDmCA

Mitochondria damage inflicted by a quinolizinium cation (**Q2**) under multiphoton irradiation at 800 nm. No damage is observed when irradiation is done in the absence of the photosensitizer quinolizinium (not shown). This movie is also available as Supplementary movie 1.

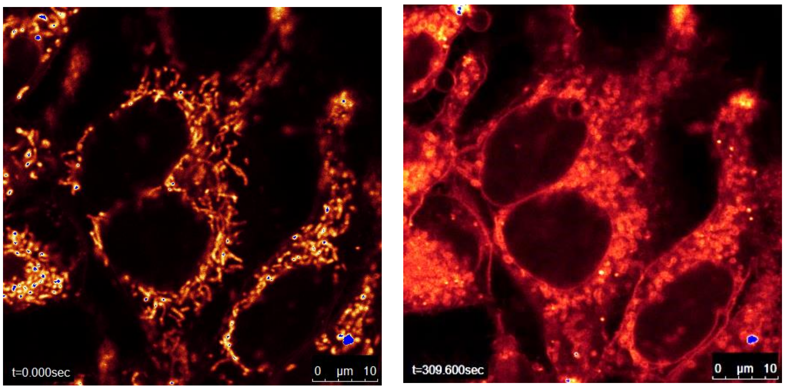

Movie with multiphoton excitation effect:
https://youtu.be/C25NQEm4ma4

Mitochondria damage inflicted by a quinolizinium cation (**Q2**) under multiphoton irradiation at 800 nm. No damage is observed when irradiation is done in the absence of the photosensitizer quinolizinium (not shown). This movie is also available as Supplementary movie 2.

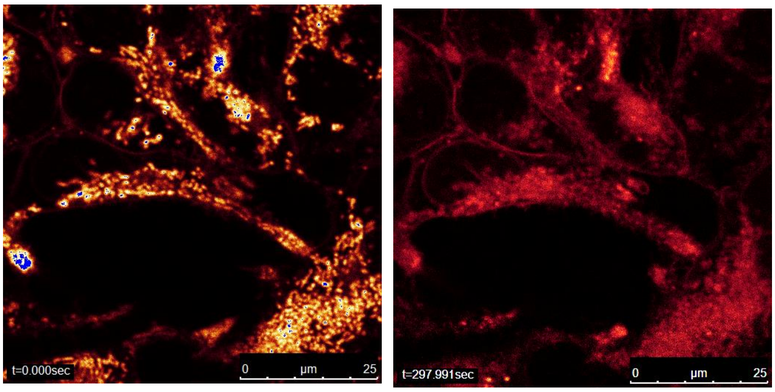

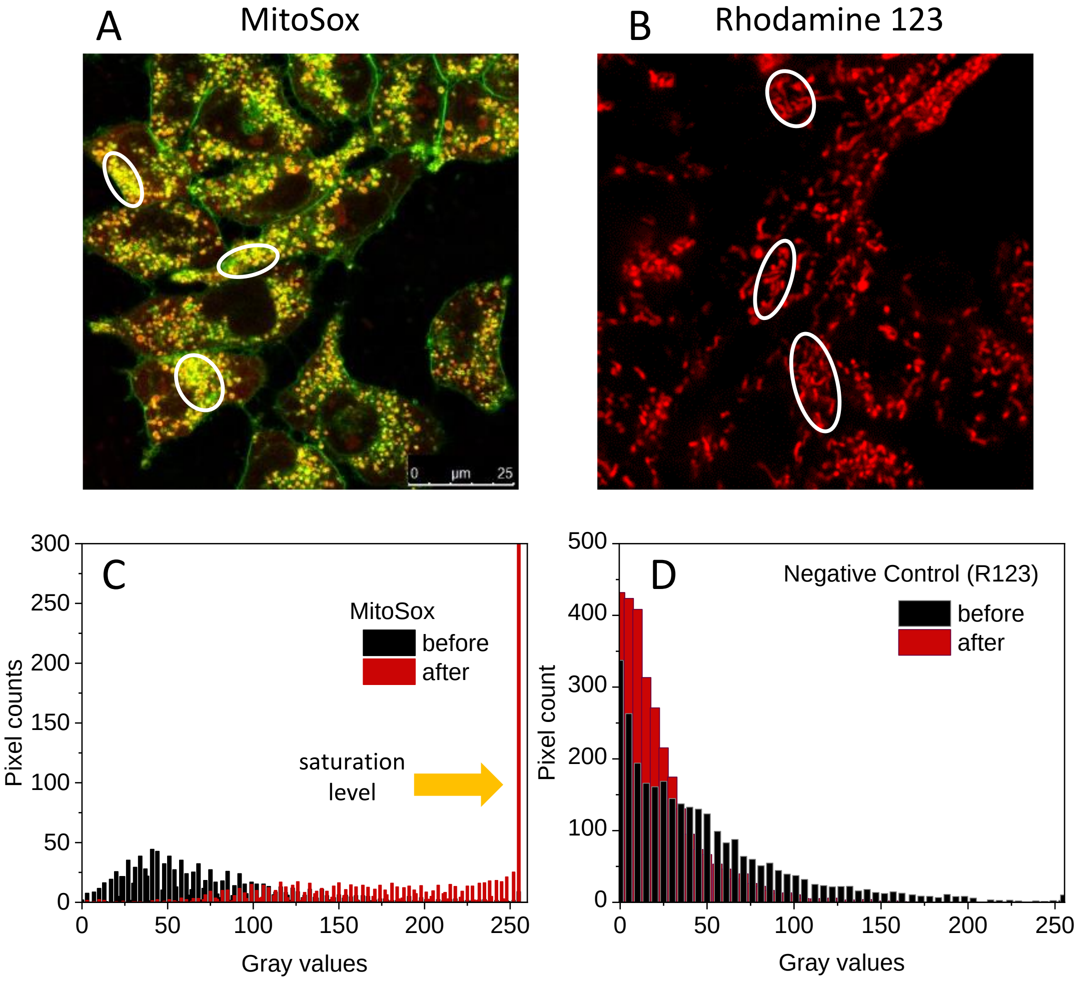

Figure S21 Effect of irradiation (800 nm, 16 mW, 20 s) on the intensity of MitoSox and Rhodamine 123 incubated in HEK293T cells. MitoSox was incubated with a **Q2** to signal the presence of superoxide. Rhodamine 123 was incubated in the absence of **Q2** to operate as a control to evaluate the effect of irradiation alone. Plots C and D show the overall distribution of pixel within the regions of interest highlighted in the corresponding images. The average pixel intensity in the MitoSox channel shown in panel C is around 41 before irradiation whereas after irradiation most of the pixels are saturated. For Rhodamine 123, the distribution of pixel intensity in panel D is only slightly shifted towards lower intensities after irradiation.

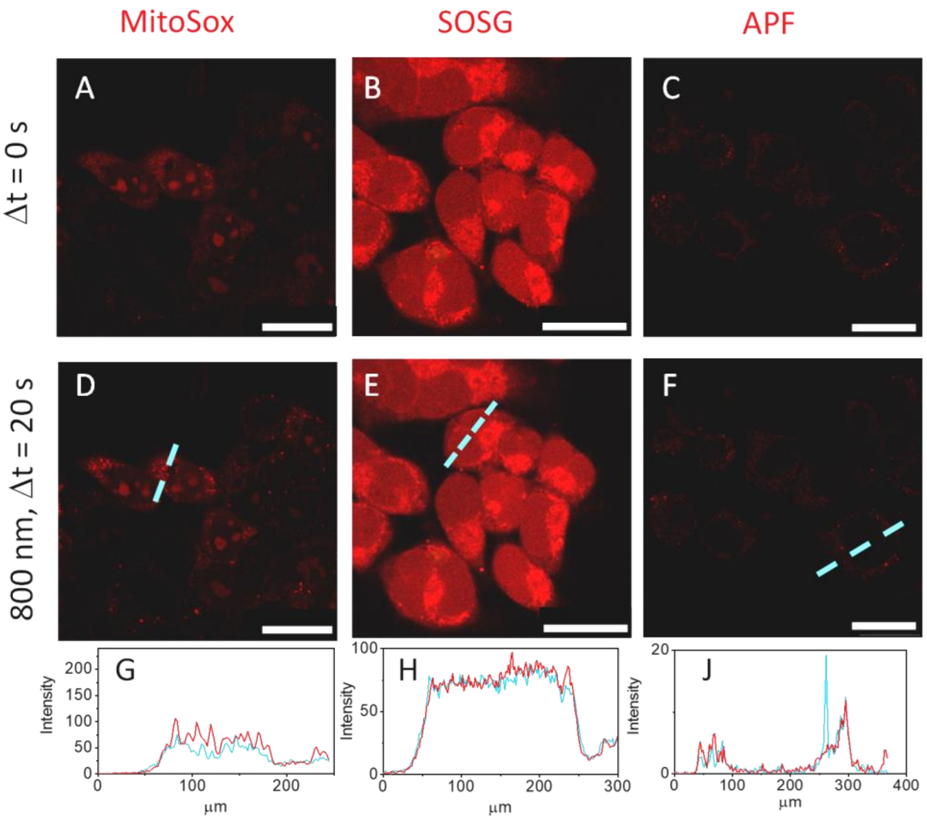

Figure S22 Control experiment showing the effect of two-photon irradiation (800 nm, 16 mW, 20 s) of HEK293T cells incubated with different ROS probes (MitoSOX, SOSG and APF) in the absence of **Q2**. Emission of the probes before and after irradiation are shown in panels A-C and D-F, respectively. The light was focused through a 63x 1.2 N.A. water immersion objective and the irradiation was done in scanning mode with 400 Hz per line at 512 x 512 pixels per scan. Fluorescence images were recorded under 514 nm excitation by collecting emission at 570-690 nm (MitoSox) or 530-650 nm (SOSG and APF). G-J are intensity profiles along the cyan dashed line in panels D-F (blue and red profiles for intensity before and after irradiation, respectively) showing that two-photon excitation did not produce any change in the intensity of the ROS probes. Scale bar equals to 25 μm.

5. Singlet oxygen quantum yield

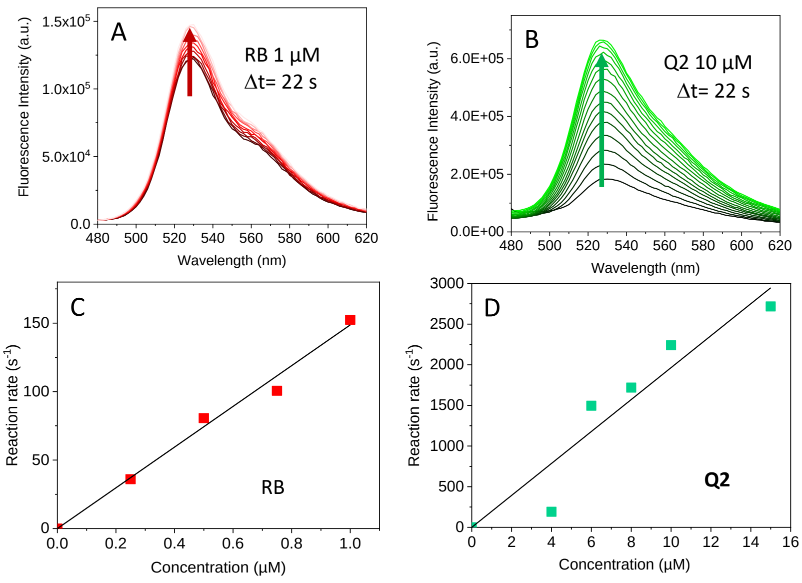

Figure S23. Quantitative measurement of $^1O_2$ generation by **Q2** using SOSG as probe and RB as a $^1O_2$ standard photosensitizer. A and B are illustrative plots of the emission of SOSG as a function of the irradiation time of RB and **Q2** at specific concentrations, with the interval between consecutive spectra determined by the time it takes to record the full spectra (22 s). C and D are the plot of the $^1O_2$ generation rate upon photosensitization of **Q2** and RB, respectively, as a function of concentration of the photosensitizer.

6. Supplementary references

1 Sato, K., Okazaki, S., Yamagishi, T. & Arai, S. The synthesis of azoniadithia 6 helicenes. *J. Heterocycl. Chem.* **41**, 443-447 (2004).

2 Caneque, T. *et al.* Novel charged NLO chromophores based on quinolizinium acceptor units. *Dyes Pigment.* **101**, 116-121 (2014).

3 Marcelo, G. *et al.* Nonlinear Emission of Quinolizinium-Based Dyes with Application in Fluorescence Lifetime Imaging. , *J Phys. Chem A* **119**, 2351-2362 (2015).

4 Maçôas, E. *et al.* A V-Shaped Cationic Dye for Nonlinear Optical Bioimaging. *Chem. Commun.* **47**, 7374-7376 (2011).

5 Cho, S. J. *et al.* Selective 5-Hydroxytryptamine 2C Receptor Agonists Derived from the Lead Compound Tranylcypromine: Identification of Drugs with Antidepressant-Like Action. *J. Med. Chem.* **52**, 1885-1902 (2009).

6 Zhou, H. P. *et al.* Fluorenylvinylenes bridged triphenylamine-based dyes with enhanced performance in dye-sensitized solar cells. *Tetrahedron* **67**, 8477-8483 (2011).

7 Zacharioudakis, E. *et al.* Quinolizinium as a new fluorescent lysosomotropic probe. *Bioorg. Med. Chem. Lett.* **27**, 203-207 (2017).

8 Benassi, R. *et al.* Conformational properties of the free and methylated 2-amino group in benzimidazole, benzoxazole, and benzothiazole - X-ray crystallographic analysis and nuclear magnetic resonance study of the internal rotation. *J. Chem. Soc.-Perkin Trans. 2*, 1513-1521 (1985).

9 Bricks, J. L., Stanova, A. V., Ryabitsky, A. B., Yashchuk, V. M. & Kachkovsky, A. D. Studies of 2-azaazulenium derivatives – 3: The nature of electron transitions and spectral properties of styryl dyes containing terminal groups of different types. **1033**, 215-226 (2013).

10 Brouwer Albert, M. in *Pure and Applied Chemistry* Vol. 83 2213 (2011).

11 Kellogg, R. E. & Bennett, R. G. Radiationless intermolecular energy transfer.3.Determination of phosphorescence efficiencies. *J. Chem. Phys.* **41**, 3042-& (1964).

12 Gaussian 09, Revision C.01, Frisch, M. J. e*t al* Gaussian, Inc., Wallingford CT, 2010.
