# Supplementary material for: Two-photon activated precision molecular photosensitizer targeting mitochondria
Source: Commun Chem. 2021 Oct 7;4:142. doi: 10.1038/s42004-021-00581-4 (PMC9814857; doi:10.1038/s42004-021-00581-4)
Supplement: Supplementary file 5 — Reporting Summary [file 42004_2021_581_MOESM5_ESM.pdf]

nature research

Corresponding author(s): Ermelinda Maçôas

Last updated by author(s): Apr 20, 2021

Reporting Summary

Nature Research wishes to improve the reproducibility of the work that we publish. This form provides structure for consistency and transparency in reporting. For further information on Nature Research policies, see our Editorial Policies and the Editorial Policy Checklist.

Statistics

For all statistical analyses, confirm that the following items are present in the figure legend, table legend, main text, or Methods section.

n/a	Confirmed	
☐	☒	The exact sample size (*n*) for each experimental group/condition, given as a discrete number and unit of measurement
☐	☒	A statement on whether measurements were taken from distinct samples or whether the same sample was measured repeatedly
☒	☐	The statistical test(s) used AND whether they are one- or two-sided<br>*Only common tests should be described solely by name; describe more complex techniques in the Methods section.*
☒	☐	A description of all covariates tested
☒	☐	A description of any assumptions or corrections, such as tests of normality and adjustment for multiple comparisons
☒	☐	A full description of the statistical parameters including central tendency (e.g. means) or other basic estimates (e.g. regression coefficient) AND variation (e.g. standard deviation) or associated estimates of uncertainty (e.g. confidence intervals)
☒	☐	For null hypothesis testing, the test statistic (e.g. *F*, *t*, *r*) with confidence intervals, effect sizes, degrees of freedom and *P* value noted<br>*Give P values as exact values whenever suitable.*
☒	☐	For Bayesian analysis, information on the choice of priors and Markov chain Monte Carlo settings
☒	☐	For hierarchical and complex designs, identification of the appropriate level for tests and full reporting of outcomes
☒	☐	Estimates of effect sizes (e.g. Cohen's *d*, Pearson's *r*), indicating how they were calculated

Our web collection on statistics for biologists contains articles on many of the points above.

Software and code

Policy information about availability of computer code

Data collection	LAS-AF (Leica), FluoroEssence (Horiba), Spectral Manager (JASCO)
Data analysis	excel, image J, origin, graphpad

For manuscripts utilizing custom algorithms or software that are central to the research but not yet described in published literature, software must be made available to editors and reviewers. We strongly encourage code deposition in a community repository (e.g. GitHub). See the Nature Research guidelines for submitting code & software for further information.

Data

Policy information about availability of data

All manuscripts must include a data availability statement. This statement should provide the following information, where applicable:

- Accession codes, unique identifiers, or web links for publicly available datasets
- A list of figures that have associated raw data
- A description of any restrictions on data availability

All data needed to evaluate the conclusions of this study are presented in this article, the Supplementary Information and/or the Supplementary Movie. Additional data related to this study are available from the corresponding authors on reasonable request.

Field-specific reporting

Please select the one below that is the best fit for your research. If you are not sure, read the appropriate sections before making your selection.

☒ Life sciences ☐ Behavioural & social sciences ☐ Ecological, evolutionary & environmental sciences

For a reference copy of the document with all sections, see nature.com/documents/nr-reporting-summary-flat.pdf

Life sciences study design

All studies must disclose on these points even when the disclosure is negative.

Sample size	N>4, in all cases we preformed at least two independent assays with two technical replicates each.
Data exclusions	No data where excluded from the analysis
Replication	We had high degree of reproducibility in our study, since in the begin of our study we took particular care in the optimization of the cell labeling protocols. We also paid particular attention to avoid mycloplasm contamination, in this sense we preformed several assays to ensured that we did not had any contamination and every month our cell culture room were clean with the Disinfectant spray "Mycoplasma-Off® mycoplasma spray". Upon optimization of the procedure, all the compounds where incubated in the cell culture at leat twice using similar incubation conditions with reproducible resullts regarding mitochondria targeting. For the ROS producton in the cell cultue, at least three replicas where performed with the different ROS probes with reproducible results. Conclusions regarding mitochondria targeting and ROS production are based on observation of a large number of cells (N>100) in each replicate. For the citotoxicty studies, we preformed at least two independent assays with two technical replicates each.
Randomization	We have studied the compounds in a single cell line, thus randomization is not relevant
Blinding	We have studied the compounds in a single cell line, thus blinding is not relevant

Reporting for specific materials, systems and methods

We require information from authors about some types of materials, experimental systems and methods used in many studies. Here, indicate whether each material, system or method listed is relevant to your study. If you are not sure if a list item applies to your research, read the appropriate section before selecting a response.

Materials & experimental systems

n/a	Involved in the study	
☒	☐	Antibodies
☐	☒	Eukaryotic cell lines
☒	☐	Palaeontology and archaeology
☒	☐	Animals and other organisms
☒	☐	Human research participants
☒	☐	Clinical data
☒	☐	Dual use research of concern

Methods

n/a	Involved in the study	
☒	☐	ChIP-seq
☒	☐	Flow cytometry
☒	☐	MRI-based neuroimaging

Eukaryotic cell lines

Policy information about cell lines

Cell line source(s)	HEK 293T human cell line . 293T cell line is a stable clone derivative of the human embryonic kidney (HEK) 293 cell line. It expresses the large T antigen of simian virus 40 (SV40).
Authentication	293T human cell line were purchased from European Collection of Authenticated Cell Cultures (ECACC)
Mycoplasma contamination	We tested our cells using the MycoAlert® Mycoplasma Detection Kit (Lonza). We did not had any contamination with mycoplasma.
Commonly misidentified lines (See ICLAC register)	no misidentification nor cross-contamination issues have been reported for the cell line used
